# Potential of Radiomics, Dosiomics, and Dose Volume Histograms for Tumor Response Prediction in Hepatocellular Carcinoma following ^90^Y-SIRT

**DOI:** 10.1007/s11307-025-01992-8

**Published:** 2025-03-10

**Authors:** Zahra Mansouri, Yazdan Salimi, Ghasem Hajianfar, Luisa Knappe, Nicola Bianchetto Wolf, Genti Xhepa, Adrien Gleyzolle, Alexis Ricoeur, Valentina Garibotto, Ismini Mainta, Habib Zaidi

**Affiliations:** 1 Division of Nuclear Medicine and Molecular Imaging, Geneva University Hospital, CH-1211 Geneva, Switzerland; 2 Service of Radiology, Geneva University Hospital, CH-1211 Geneva, Switzerland; 3 Centre for Biomedical Imaging (CIBM), Geneva, Switzerland; 4 Laboratory of Neuroimaging and Innovative Molecular Tracers (Nimtlab), Geneva University Neurocenter and Faculty of Medicine, University of Geneva, Geneva, Switzerland; 5 Department of Nuclear Medicine and Molecular Imaging, University of Groningen, University Medical Center Groningen, Groningen, Netherlands; 6 Department of Nuclear Medicine, University of Southern Denmark, Odense, Denmark; 7 University Research and Innovation Center, Óbuda University, Budapest, Hungary

**Keywords:** SIRT, Radiomics, Dosiomics, Dose–response effect, ^90^Y-SIRT, Machine learning

## Abstract

**Purpose:**

We evaluate the role of radiomics, dosiomics, and dose-volume constraints (DVCs) in predicting the response of hepatocellular carcinoma to selective internal radiation therapy with ^90^Y with glass microspheres.

**Methods:**

^99m^Tc-macroagregated albumin (^99m^Tc-MAA) and ^90^Y SPECT/CT images of 17 patients were included. Tumor responses at three months were evaluated using modified response evaluation criteria in solid tumors criteria and patients were categorized as responders or non-responders. Dosimetry was conducted using the local deposition method (Dose) and biologically effective dosimetry. A total of 264 DVCs, 321 radiomic features, and 321 dosiomic features were extracted from the tumor, normal perfused liver (NPL), and whole normal liver (WNL). Five different feature selection methods in combination with eight machine learning algorithms were employed. Model performance was evaluated using area under the AUC, accuracy, sensitivity, and specificity.

**Results:**

No statistically significant differences were observed between neither the dose metrics nor radiomicas or dosiomics features of responders and non-responder groups. ^90^Y-dosiomics models with any given set of inputs outperformed other models. This was also true for ^90^Y-radiomics from SPECT and SPECT-clinical features, achieving an AUC, accuracy, sensitivity, and specificity of 1. Among MAA-dosiomic and radiomic models, two models showed AUC ≥ 0.91. While the performance of MAA-dose volume histogram (DVH)-based models were less promising, the ^90^Y-DVH-based models showed strong performance (AUC ≥ 0.91) when considered independently of clinical features.

**Conclusion:**

This study demonstrated the potential of ^99m^Tc-MAA and ^90^Y SPECT-derived radiomics, dosiomics, and dosimetry metrics in establishing predictive models for tumor response.

**Supplementary Information:**

The online version contains supplementary material available at 10.1007/s11307-025-01992-8.

## Introduction

Selective internal radiation therapy (SIRT) using ^9^^0^Y-microspheres is one of the well-established and effective treatment options for unresectable liver malignancies, such as hepatocellular carcinoma (HCC) as the most common type of primary liver cancer. The treatment procedure involves simulating the distribution of ^90^Y microspheres within the tumoral and healthy regions of the liver. This is achieved using ^99m^Tc-macroaggregated albumin (^99m^Tc-MAA) as a surrogate, providing a predictive model of microsphere deposition and ensuring accurate targeting while minimizing damage to healthy tissue. ^99m^Tc-MAA is also used for personalized pre-therapeutic dosimetry and estimating the shunts to other organs, such as the lungs [1].

One of the emerging, yet well-studied paradigms that can profile intra-tumor heterogeneities, subsequently providing valuable information for personalized therapy, is radiomics [2]. Radiomics is an advanced quantitative image analysis tool, involving the extraction of quantitative features and utilizing them for developing task-dependent models that can be eventually used for clinical decisions [2–5].

Several studies have demonstrated the potential of radiomics to predict tumor response to ^90^Y-SIRT [6–9]. Wei et al. [6] used post-treatment ^90^Y-PET radiomic features extracted from liver lesions to predict tumor treatment response and reported an area under the receiver operating (ROC) curve (AUC) of 0.713. Ince et al. [7] employed pre-treatment MRI radiomics features alongside clinical features. Their study demonstrated superior performance of models utilizing radiomic features compared to those relying solely on clinical models. Marinelli et al. [8] used post-treatment MRI radiomics features extracted from tumors with the aim of early prediction of treatment response. The reported AUC was 0.89.

Dose-volume histograms (DVHs) are reliable tools commonly used for reporting the absorbed dose received by specific volumes. DVHs provide a quantitative summary of dose distribution through visual depiction and facilitate the comparison of two different dose distributions [10, 11]. The capability of DVH-based dosimetry parameters in predicting time-to-event survival has been demonstrated for ^90^Y-SIRT [12].

The concept of extracting radiomic features from dose distributions, known as dosiomics, enables the characterization of spatial heterogeneity within dose maps [13–16]. Dosiomics can be used as a complementary approach to radiomics for profiling both tumor and absorbed dose heterogeneities. There is an indication that dosiomics provides a more comprehensive depth of insights compared to conventional DVH calculations [14].

However, radiomics, and consequently dosiomics, involve multi-step processes, each with their own challenges related to machinery, operations, and analytical procedures, which can impact the reproducibility and repeatability of these features. As such, errors may be present in the results [17]. Incorporating these techniques into patient management processes requires additional calculations while DVH calculation does not need complex computations and is readily available on most treatment planning systems. In addition, dose-volume constraints (DVCs) that are defined to make a compromise between treatment effectiveness and toxicities, convey useful information that may be related to treatment response and outcome prediction [12].

This study aimed at assessing the feasibility and significance of utilizing radiomics, dosiomics, DVH-driven parameters, and clinical biomarkers to predict tumor response to ^90^Y-SIRT. It is a preliminary, yet comprehensive investigation that involves extracting radiomic features from both ^99m^Tc and ^90^Y SPECT and co-registered CT images. Additionally, physical and biologically effective dosimetry (BED) maps from both planning and treatment procedures were used for dosiomics assessment. Furthermore, routine DVCs in personalized SIRT treatment were extracted from physical and BED DVHs and were utilized to explore if these parameters play a role in tumor response prediction. The features were employed in distinct strategies to develop machine learning models for tumor response prediction. These methodologies were formulated to address potential data gaps in clinical setting, ensuring that even in the clinics where specific data or calculations might be lacking, there is still a feasible model that can be utilized.

## Materials and Methods

### Patient Cohort

SIRT with personalized dosimetry was conducted for 28 patients with different types of liver cancers, utilizing ^90^Y bremsstrahlung SPECT/CT imaging, at Geneva University Hospital, Switzerland, between November 2021 and January 2023. This retrospective stu*d*y was approved by the institutional ethics committee and the requirement to obtain informed consent was waived. Among this number of patients, 17 were treated for hepatocellular carcinoma (HCC) tumors and were included in this retrospective study. Glass microspheres (Therasphere™; Boston scientific group, Marlborough, Massachusetts) were used for treatment. Selection criteria for SIRT included: Adult patients with at least one well-defined tumor > 3 cm, stable liver enzymes, no contraindications to angiography, no concurrent treatment, no previous transplantation, or previous liver resection and an Eastern Cooperative Oncology Group (ECOG) performance status of 0 to 1. Patient characteristics are summarized in Supplemental Table 1. The Mann–Whitney U test and Fisher’s Exact test were used to compare the clinical continuous and categorical features between responder and non-responder groups, respectively.

### ^90^Y-SIRT Treatment

After mapping the liver vessels and evaluating the extrahepatic shunts, personalized voxel-level dosimetry and treatment planning were performed using ^99m^Tc-MAA SPECT/CT imaging for each patient to simulate the therapy. Lung shunt fraction (LSF) was calculated based on planar images. Post-treatment absorbed dose distributions were calculated through ^90^Y bremsstrahlung SPECT/CT imaging. Simpliciti90Y™ (Mirada Medical Ltd, United Kingdom) treatment planning system was used for dosimetry calculations. The median of ^99m^Tc-MAA injected activity was 156 ± 9.7 MBq, whereas the median of ^90^Y injected activity was 2.8 ± 1.17 GBq. The initial activity was calculated using the partition model specified for each individual.

### Image Analysis

Baseline diagnostic images were acquired either by multi-phasic contrast-enhanced CT (Siemens Healthineers, SOMATOM, Erlangen, Germany) at 100 kVp and the tube current of 315.2 ± 216.71 mA (average ± SD), or 3T MRI (Siemens healthineers, Erlangen, Germany). SPECT/CT images were acquired on a dual-head Symbia-T series camera (Siemens Healthineers, Erlangen, Germany) using a low-energy high-resolution collimator. The energy window center of ^99m^Tc SPECT was set to 140 keV [128–150]. A matrix size of 128 × 128, with 128 views, over a 360-degree arc and 20–25 s per view were utilized. The bremsstrahlung SPECT/CT scans were acquired under a continuous energy window [105–195] keV using high energy collimators, and 128 frame with 15–30 s per frame. SPECT/CT data were reconstructed using a 3D ordered-subset expectation maximization (3D-OSEM) algorithm with 4 iterations and 8 subsets. Reconstruction was performed with attenuation correction but without scatter correction followed by 5mm Gaussian post-reconstruction filtering.

The follow-up (FU) contrast-enhanced CT was acquired on SOMATOM scanner (Siemens Healthineers, Erlangen, Germany) 1 month and every 3 months post-therapy mostly with 100 and 120 kVp and 352.14 ± 135 mA (average ± SD) tube current. If CT images at a specific timepoint were not acquired, 3T MR images (Siemens healthineers, Erlangen, Germany) were collected. Tumor boundaries were localized, and target volumes (tumors) delineated on arterial phase of baseline and FU images by an experienced nuclear medicine physician and gastrointestinal (GI) interventional radiologists who were blinded to clinical, biological, survival, and dosimetry data. To assess the outcome, tumor response was evaluated up until 3 months post-treatment based on modified response evaluation criteria in solid tumors (mRECIST) comparing relative changes in tumor largest diameter on axial view of CT or MRI (if CT was unavailable) [18]. Based on the response to treatment, patients were categorized as responders (R) (including complete response [CR] meaning disappearance of any intertumoral arterial enhancement in all target lesions, and partial response [PR] groups meaning at least a 30% decrease in the sum of the diameters) and non-responders (NR) (including stable disease [StD] meaning insufficient shrinkage to qualify for PR and insufficient increase to qualify for progressive disease [PD], and PD groups, meaning an increase of at least 20% in the sum of the diameters of viable target lesions) by GI interventional radiologists.

### Dosimetry Calculations

After tumor manual delineation by a nuclear medicine physician on diagnostic images, tumor segmentations were transferred onto SPECT/CT images by registration of diagnostic images and co-registered CT of SPECT/CTs performed using elastix library and a two-step rigid and deformable registration using mutual information similarity metric [19]. The registration step was performed using rigid-body registration followed by deformable algorithms. The perfused lobe and whole liver were delineated on the co-registered ACCT of SPECT/CT images by a physician and a previously trained deep learning model [20], respectively. Normal structures namely, normal perfused liver (NPL) and whole normal liver (WNL) were obtained by subtracting the tumor from the perfused lobe and whole liver.

The physical as well as the biologically effective dose (BED) distributions were calculated for both ^99m^Tc and ^90^Y SPECT of each patient. An in-house MATLAB code (MATLAB (2022b), Natick, Massachusetts: MathWorks Inc) was validated against replicated analysis with Simplicit90Y™ (Boston Scientific, Marlborough, MA) and was utilized to calculate the 3D voxel-level physical dose maps based on local energy deposition method. 3D voxel-level BED maps were also calculated for tumoral and normal structures separately using the following formula:

1 $$BED=D\left(1+\frac{D}{{~}^{\alpha }\!\left/ \!{~}_{\beta }\right.} . \frac{{T}_{Rep}}{{T}_{Rep}+{T}_{phys}}\right)$$

where D is the cumulative dose of ^90^Y radiation, T_Rep_ is the sublethal damage repair half-time and T_phys_ is the radionuclide decay half-life (64.2 h). α⁄β ratios and T_Rep_ for Tumor and normal tissue are derived from Chiesa et al. study [21]. The designated values for α⁄β were 10, while T_Rep_ (h) was set to 1.5 and 2.5 for Tumoral and Normal structures, respectively. The DVC parameters extracted from ^99m^Tc and ^90^Y DVHs and biologically effective dose-volume histograms (BVHs) for each structure are listed as follows:

The evaluated parameters include the volume of structures, mean absorbed dose, maximum dose, minimum dose, D_50_, D_70_, D_95_, D_98_, V_120_, V_205_, and V_400_ for all structures. Additionally, for NPL and WNL, V_20_, V_30_, V_50_, V_70_, and V_90_ were also assessed. All volume-based constraints (V_20_-V_400_) were calculated using both milliliter (ml) and percentage (%) units.

Additionally, we computed other features, such as a simplified version of the tumor dose homogeneity index (HI = D_5_/D_95_), commonly used in external beam radiotherapy (EBRT). Furthermore, we calculated the tumor to normal liver ratio (TNR) with respect to both NPL (TNR_NPL_) and WNL (TNR_WNL_) for both ^99m^Tc and ^90^Y procedures. Two other (personalized) dosimetry-related parameters, including LSF, and ^90^Y-injected activity were also utilized as predictive features. A Mann–Whitney U-test was conducted to compare the dose values between the R and NR groups.

### Radiomic and Dosiomic Feature Extraction

The radiomic features were extracted from co-registered CTAC and SPECT images while dose maps, whether physical or BED maps, were utilized for extracting dosiomic features. CT images were resampled to 1.5 × 1.5 × 1.5 mm^3^ and clipped between −500 to 500 HU prior to feature extraction. SPECT images were clipped between 0 and 99.9th percentile of the counts/s values for each image. Dose maps were clipped within the range of 0 to the median of 95th percentile of the dose values (Gy) across all patients, varying based on the anatomical structure and radionuclide used, i.e., fixed values were used for all patients. For ^99m^Tc dose maps, these values were 650.34, 158.02, and 127.08 Gy for the tumor, NPL and WNL, respectively. Similarly, for ^90^Y dose maps, the corresponding values were found to be 416.25, 133.16, and 123.08 Gy for the tumor, NPL and WNL, respectively. For extracting the features, the bin width was set to 50 counts/s, 20 HU, and 1 Gy for SPECT, CT, and dose maps, respectively. These bin width values were selected to make a compromise between keeping the valuable information and computational effort. For example, the bin width of the dose maps was selected to be 1 Gy to keep the details of dosimetry calculations while maintaining an efficient computational burden and time.

A total of 321 features were calculated for all three structures (tumor, NPL and WNL), comprising 107 features each, using pyradiomics library (version 3.1.0) [22]. Each set of features consists of 19 First-Order Statistics, 16 Shape-based (3D), 10 Shape-based (2D), 24 Gray Level Co-occurrence Matrix, 16 Gray Level Run Length Matrix, 16 Gray Level Size Zone Matrix, 5 Neighboring Gray Tone Difference Matrix, and 14 Gray Level Dependence Matrix features. The features for all structures were combined and used in the next steps.

### Strategy Devising

We devised multiple strategies to comprehensively evaluate all potential scenarios:

1. Radiomics from ^99m^Tc MAA SPECT or CT images,
2. Radiomics from ^90^Y SPECT or CT images,
3. Dosiomics from MAA physical or BED dose maps,
4. Dosiomics from ^90^Y physical or BED dose maps,
5. DVH-driven parameters from MAA dose maps,
6. DVH-driven parameters from ^90^Y dose maps.

Each of these six categories encompasses six subcategories, including:

#### DVH and Dosiomics

- Physical dose denoted as “Dose”
- BED
- Dose + BED
- Dose + Clinical
- BED + Clinical
- BED + Dose + Clinical

#### Radiomics

- CT
- SPECT
- CT + Clinical
- SPECT + Clinical
- CT + SPECT
- CT + SPECT + Clinical

This results in a total of 36 different strategies. In addition, 16 clinical features were used which are indicated in Supplemental Table 1, among them sex and extrahepatic metastasis (only one patient had metastasis) were not used because of extreme unbalance between R and NR groups. A Mann–Whitney U-test was also conducted to compare the radiomics and dosiomics feature values between the R and NR groups. The test was followed by a Benjamini and Hochberg (BH) test with q = 0.05 to adjust the p-values and find the false discovery rate.

### Feature Selection and Machine Learning Modeling

To train the models, we employed a threefold nested cross-validation (CV) approach, comprising an inner and an outer CV loop, to prevent overfitting and ensure a more reliable performance. Features extracted from training sets of each strategy were normalized to their Z-score, with the resulting mean and standard deviation applied to corresponding features extracted from the test datasets within the outer loop.

Different machine learning (ML) algorithms combined with different feature selection (FS) methods, aiming at identifying the most relevant features and eliminating redundant ones, were utilized. ML modeling was carried out using eight different algorithms, including Decision Tree (DT), Generalized Linear Mixed Model Boosting (GLMB), Logistic Regression (LR), Multiple Layer Perceptron (MLP), Naïve Bayes (NB), Random Forest (RF), Support Vector Machine (SVM), and Extreme Gradient Boosting (XGB). We used five different FS methods, including ANOVA, Kruskal, Minimum Redundancy Maximum Relevant (MRMR), Randomized Ensemble Feature Importance (Relief), and Recursive Feature Elimination (RFE). The redundant features were removed using Spearman's rank correlation coefficient. A rho of 0.90 was used as threshold.

Hyperparameter optimization was carried out using Grid Search with threefold cross-validation within the inner CV loop, and the best values employed for model training. Given the small sample size, we also generated 1000 bootstrap samples with replacement for ROC curves. The dataset was imbalanced between the number of treatment non-responders and responders. Synthetic Minority Oversampling Technique (SMOTE) was used on the training sets to overcome any biases in model performance due to unbalanced dataset. SMOTE was used during hyperparameter optimization on the inner training dataset, and once the best hyperparameters were chosen, it was also applied to the outer training dataset. The final trained model was evaluated on the outer test dataset. Eventually, we ended up trying 1440 models (resulting from 8 ML × 5 FS × 6 categories × 6 subcategories). Supplemental Table 2 summarizes the hyperparameters and their ranges for each classifier.

**Fig. 1:**
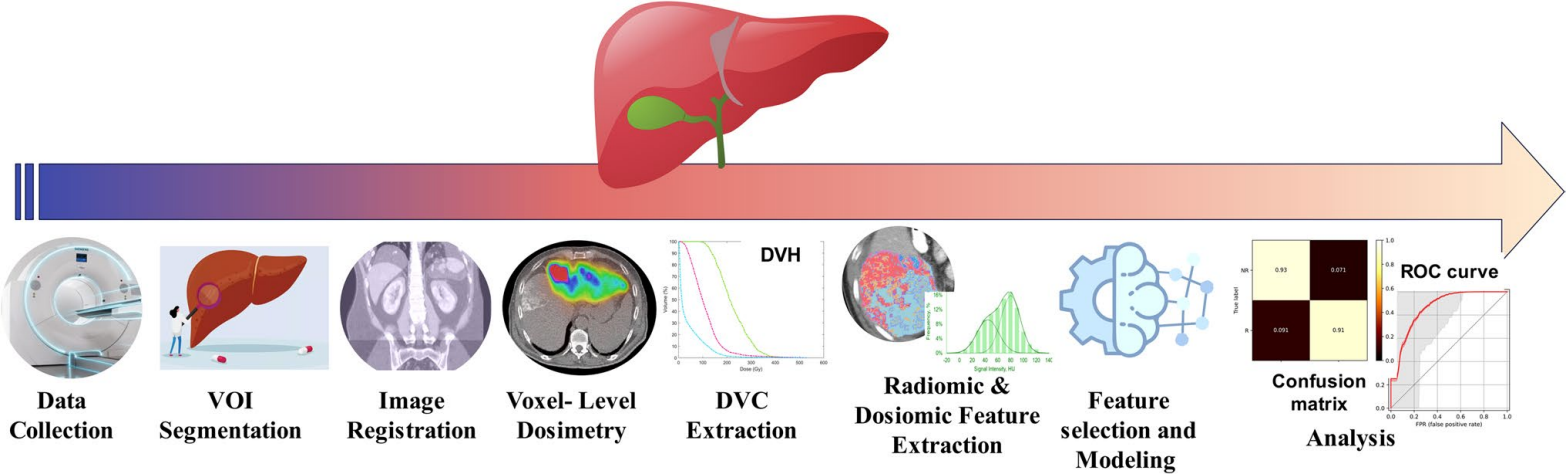
Flowchart depicting the procedural steps followed in this study. VOI: volume of interest, DVC: dose-volume constraint

**Fig. 2:**
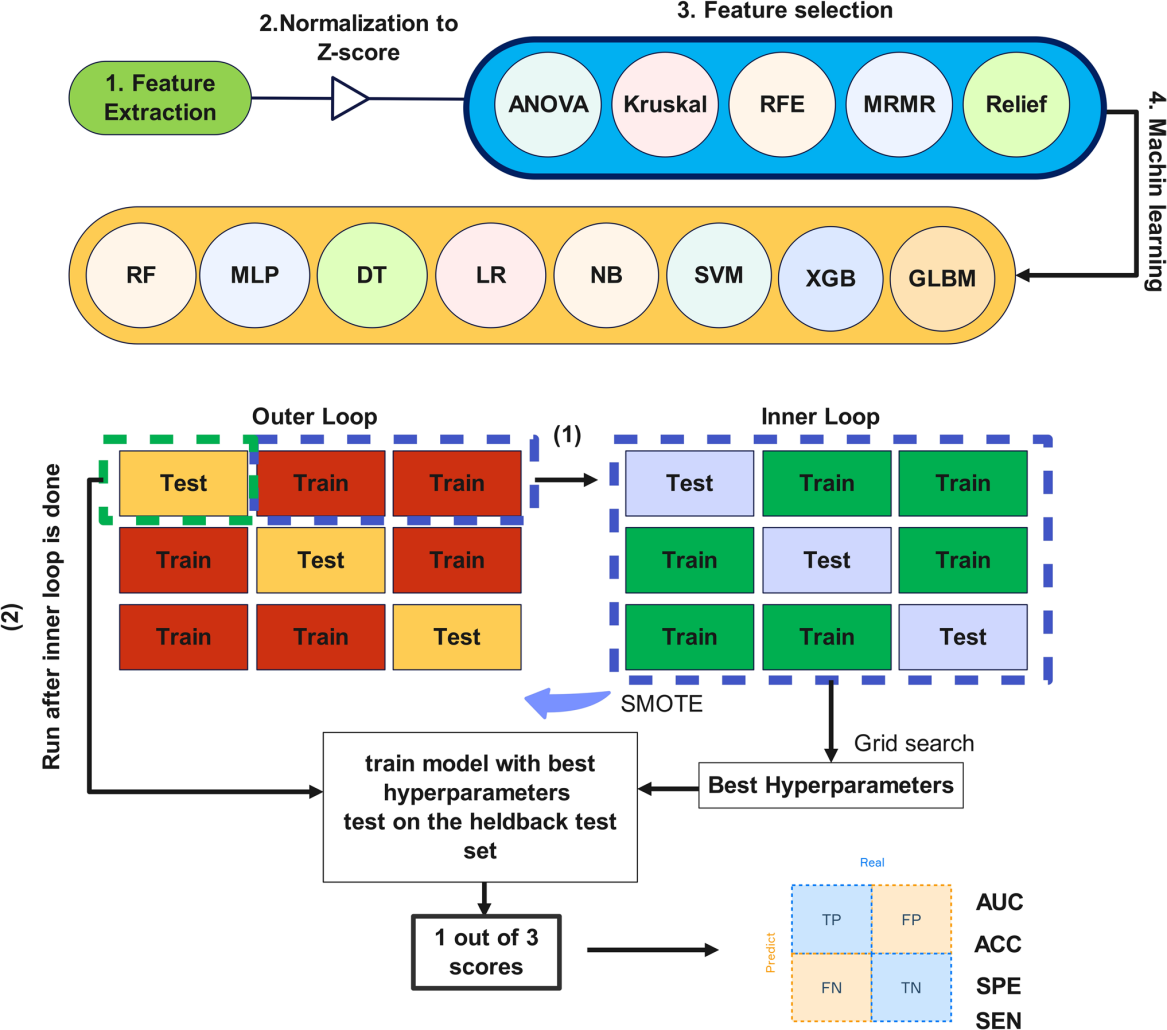
The machine-learning procedure and 3-fold nested cross-validation concept. After feature extraction and normalization, five different feature selection methods were used to eliminate the redundant features. The selected features were used for machine-learning procedures with eight different algorithms in a 3-fold nested cross validation framework. The AUC, Accuracy (ACC), Specifity (SPE) and sensitivity (SEN) were calculated

For every model, a confusion matrix was computed, detailing true negative (TN), true positive (TP), false negative (FN), and false positive (FP) rates. Model performance was assessed using metrics, such as Area Under the Receiver Operating Characteristic Curve (AUC), accuracy (ACC), sensitivity (SEN), and specificity (SPE). To ascertain the most robust models, Delong statistical test was employed to compare AUC values, with a P-value < 0.05 indicating statistical significance. Figure 1 outlines the study's flowchart whereas Fig. 2 summarizes the machine learning procedure.

## Results

### Tumor Response and Dosimetry

**Fig. 3:**
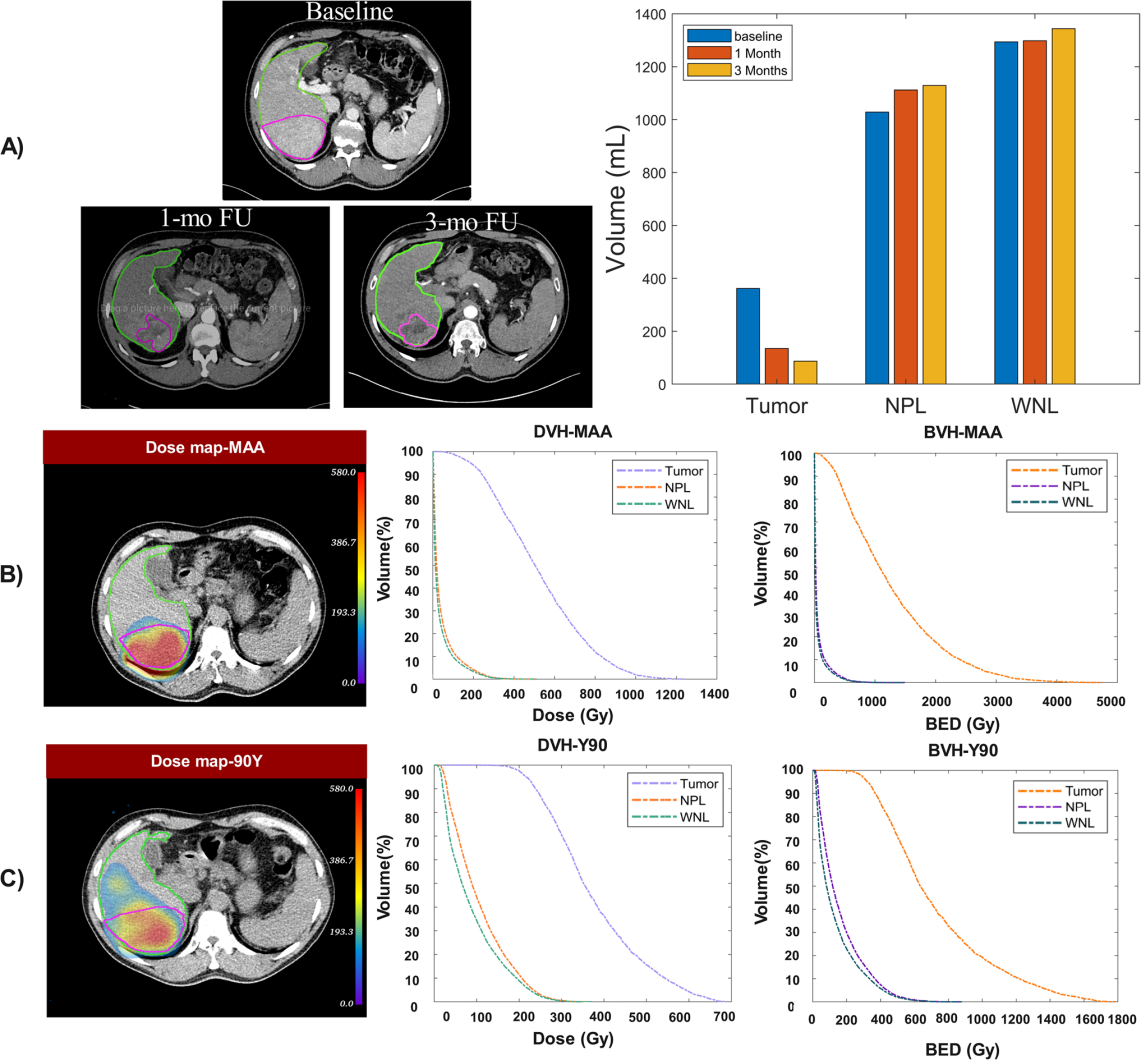
Representative images and absorbed dose metrics of a patient with partial response. **A** from left to right, baseline CT, follow-up (FU) CT one-month post-therapy, and 3 months follow-up CT. The lesion and liver are outlined in magenta and green, respectively. The bar plot shows the changes in tumor, normal perfused liver (NPL) and whole normal liver (WNL) volumes. **B** ^99m^Tc-MAA dose map and corresponding DVH and BVH plots. **C** ^90^Y dose map with corresponding DVH and BVH plots

The tumor response of 17 patients was evaluated at three months post-therapy according to the mRECIST criteria. No complications were reported during the follow-up. Five tumors were classified as responders (R), while 12 were non-responders (NR). Within the NR group, 11 tumors showed stable disease (StD), and one case showed progressive disease (PD). Similarly, in the R group, five cases of partial response (PR) and no case of complete response (CR) were observed. Figure 3 shows a representative case demonstrating PR and the changes in tumor and normal tissue volumes. The figure also illustrates dose maps from ^99m^Tc-MAA and ^90^Y in the tumor and normal tissue volumes, along with corresponding DVHs and BVHs.

Statistically significant differences were neither observed between mean absorbed doses and mean BEDs in NR and R groups, nor in other DVC metrics (p-value > 0.05). The mean absorbed dose values for the case demonstrating partial response were 345.6 ± 121.7 and 734.73 ± 356.31 Gy for mean dose and mean BED from ^99m^Tc-MAA dose maps, respectively. Meanwhile, the corresponding values for ^90^Y treatment were 244.7 ± 83.28 and 413.78 ± 194.4 Gy for mean dose and mean BED, respectively. Supplemental Table 3 represents the dosimetry parameters for R and NR groups.

### Radiomic and Dosiomic Features

**Fig. 4:**
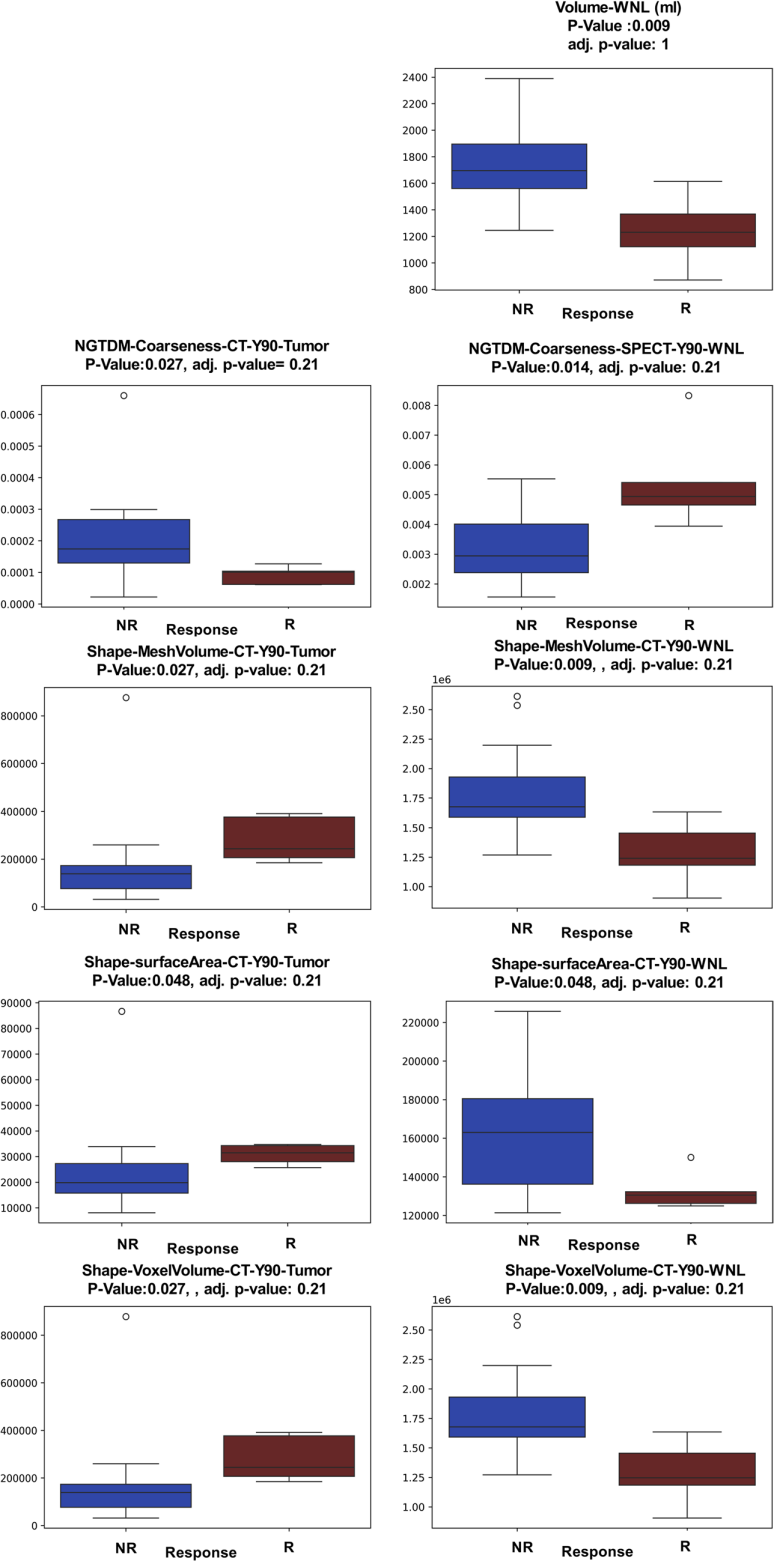
Examples of features with statistically significant differences (with the initial Mann–Whitney U-test) between the NR and R groups. All features were calculated using ^90^Y CT scans. The texture feature (NGTDM-Coarseness) and three other shape-based features demonstrated opposite behaviors for tumors and WNL. For instance, one feature might be higher in the R group compared to the NR group for tumors, while showing an opposite trend for WNL. These features however recognized as not statistically significant following a Benjamini and Hochberg complementary test

The features were compared statistically between the R and NR groups. The results from the Mann–Whitney U-test indicated significant differences between the two groups for many features. However, we performed Benjamini and Hochberg (BH) test with q = 0.05 to find the false discovery rate. According to the results of this test, none of the features showed statistically significant associations. As examples, Fig. 4 presents boxplots as an example, showing the distributions of feature values with statistically significant differences between the NR and R groups.

In Fig. 4, we highlighted the Neighborhood Gray-Tone Difference Matrix-Coarseness (NGTDM-Coarseness) texture feature and three other shape-based features, which exhibited opposite behaviors for tumors and WNL. Specifically, while one feature might demonstrate higher values in the R group compared to the NR group for tumors, it could display the opposite trend for WNL. For example, the NGTDM-Coarseness, a texture feature from ^90^Y-CT is higher for the tumors of NR and for the WNL of R. The other three features are shape-related: "Shape-Mesh-Volume", "Shape-Surface-Area", and "Shape-Voxel-Volume", which have higher values in the tumors of R and the WNL of NR.

**Fig. 5:**
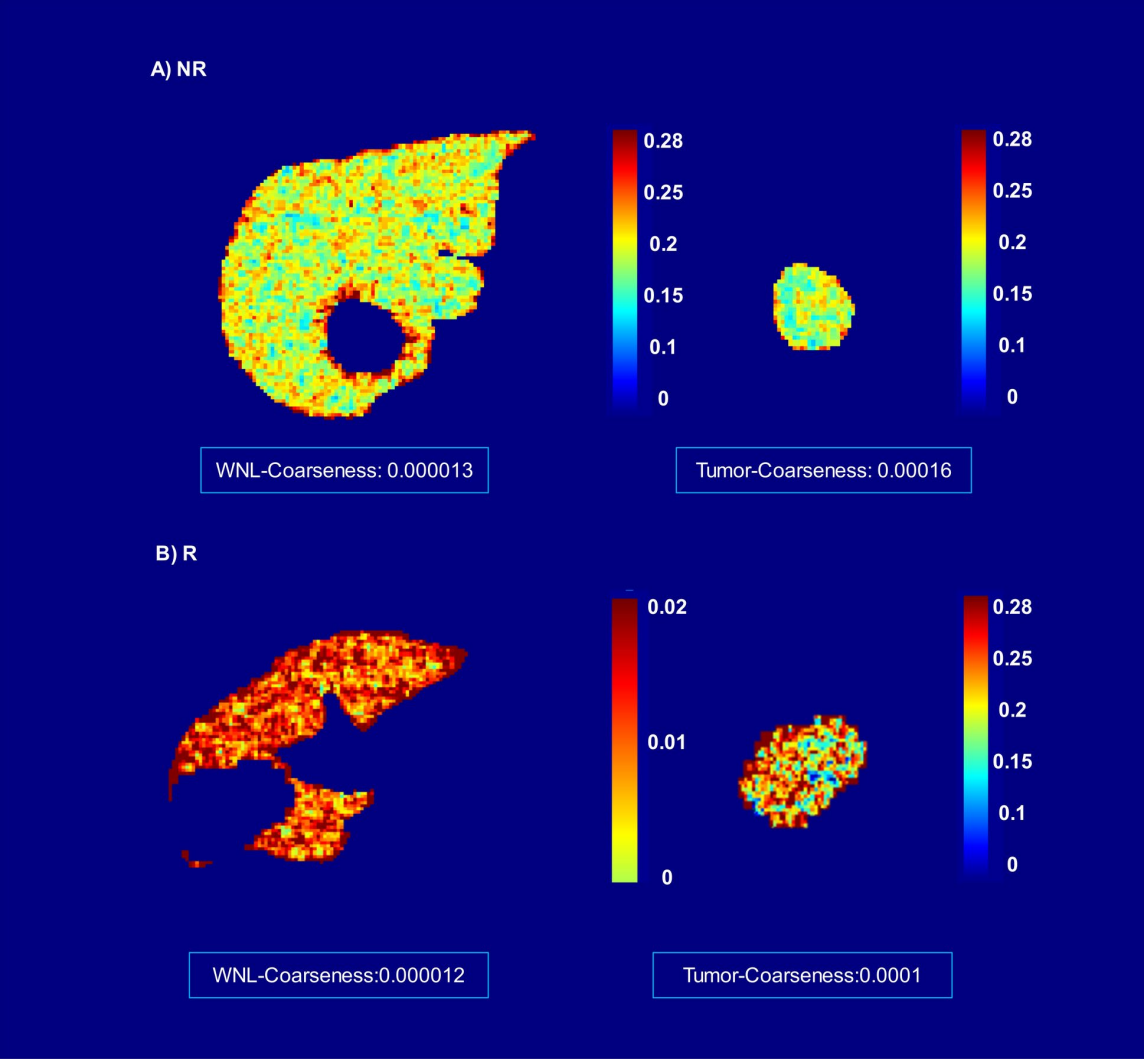
The NGTDM-Coarseness feature maps show different patterns for non-responder (NR) and responder (R) cases. **A** In the NR case, the feature value for the whole normal liver (WNL) is lower than that for the tumor, indicating that intensity values are smoother in the tumor and more variable in the WNL. **B** Conversely, in the R case, the opposite pattern is observed

Figure 5 displays an example of the feature maps calculated for NGTDM-Coarseness texture feature. This feature quantifies the spatial variation of gray-level intensities within a region of interest (ROI). A higher value of this feature indicates greater uniformity (smoother) in intensity across the ROI.

The NGTDM-Coarseness feature was among the most frequently selected features using the Kruskal FS method, with high importance values (NGTDM-Coarseness-Tumor = 6.5 and NGTDM-Coarseness-WNL = 5.04) in models where CT-radiomic features were included. This feature was also identified as a robust radiomic feature extracted from PET images in a phantom study [6].

### Machine Learning Models

We selected models that demonstrated superior performance in predicting tumor response, each trained with distinct inputs from various strategies. The selected models, along with their corresponding inputs and performance evaluation metrics, are summarized in Table 1. Supplemental Fig. 1 shows the heatmaps for AUC, Accuracy, Sensitivity and Specificity. The results of Delong tests, which assess the statistical significance of models within each strategy are provided in Supplemental Fig. 2.

**Fig. 6:**
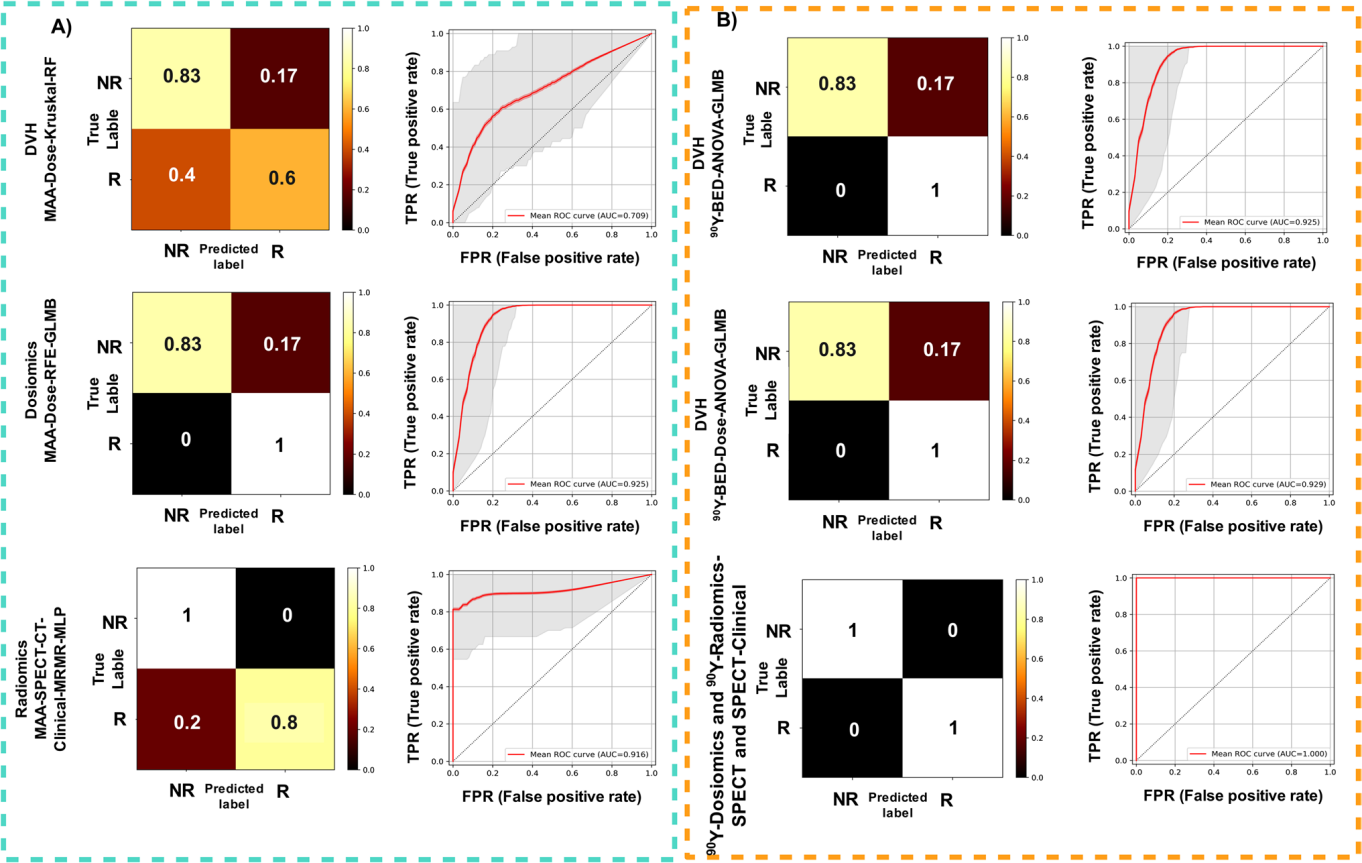
The confusion matrices and corresponding ROC curves for well-performing models across various strategies, trained on two datasets: **A** ^99m^Tc-MAA dataset and **B** ^90^Y dataset. The AUC values for ^90^Y models were higher than those for the ^99m^Tc-MAA models. In panel B, many of the Dosiomics and Radiomics models achieved an AUC of one. Therefore, we display only one ROC curve and confusion matrix as a representative for all

Figure 6 shows the confusion matrices and ROC curves of selected models for both ^99m^Tc-MAA and ^90^Y. As the statistical test did not reveal any significant difference among the selected models for DVH, radiomics and dosiomics strategies, we only displayed the ROC curve and confusion matrix for the models with the highest AUCs in each category for clarity.

A list of selected features contributing to model training are provided in Supplemental Table 4. To further emphasis explainability of the models, we also demonstrated the feature importance of the selected features in Supplemental Fig. 3. For clarity only the features selected in SPECT and SPECT-Clinical radiomics models (AUC = 1) which outperformed the other radiomics models are presented here. The feature importance of features contributing to dosiomics, and DVH-based models are presented in Supplemental Fig. 4.

## Discussion

Our study represents a significant advancement in the evaluation of tumor response by integrating multivariate radiomics and dosiomics analyses. Notably, to our knowledge, this is the first study evaluating dosiomics computed from both ^99m^Tc- and ^90^Y-SPECT-related absorbed dose maps. Additionally, we pioneered the use of DVH-based dosimetry variables as features to train models for tumor response prediction.

Inspired by recent research emphasizing the importance of Organ-at-Risk (OAR) radiomics in predicting overall survival [23], we extended our analysis to include features extracted from OARs, such as NPL and WNL. Surprisingly, our results indicated that features extracted from these OARs were as important as those extracted from the tumor itself. Among the OAR features that were used for training, several were frequently selected, particularly those from WNL.

For response evaluation, we used mRECIST criteria, which assesses viable tumors delineated on the arterial phase of contrast-enhanced imaging techniques [18]. Currently, there is no standardized functional imaging for HCC, and as such, response assessment relies on anatomical methods, such as RECIST, World Health Organization (WHO) criteria, European Association for the Study of the Liver (EASL) criteria, and mRECIST criteria [24]. RECIST and WHO criteria are primarily designed for systemic therapies and have limitations when applied to locoregional therapies like SIRT. In contrast, EASL and specifically mRECIST criteria are more commonly used for evaluating the effectiveness of SIRT [25–28]. The limitations of tumor assessment in this study arise from the inherent assumptions and constraints of the mRECIST criteria, which presume spherical and symmetrical tumor shrinkage—an assumption that is not always valid [29].

Personalized dosimetry plays an important role in improving the treatment outcomes in HCC patients [30, 31]. One of the strengths of this study is that we utilized data from patients who underwent personalized planning prior to SIRT. Moreover, we calculated BED maps. BED calculations allow for radiation-based combination therapies and facilitate comparisons between EBRT and SIRT, despite the fundamental differences between these two treatment modalities [32, 33]. BED represents the dose of a single source of ionizing radiation needed to achieve the same biological effect as a reference irradiation. The calculation of the BED was based on α/β values derived from tumor control probability model specifically designed for a glass microsphere SIRT study [21]. However, most studies typically adopt these values from EBRT, despite the fundamental differences between EBRT and SIRT. Further investigations into BED calculations for SIRT are necessary.

SPECT imaging voxel values in “counts per second”, or “total counts collected” unit can vary with the acquisition duration, injected activity, and time interval between the injection and imaging. The local energy deposition method involves a step for self-calibration, which is helpful in moving toward quantification. Both ^99m^Tc-MAA and ^90^Y Therasphere have a long biological half-life when injected in the hepatic artery. This fact allows to use the whole patient’s body (or the whole liver) as a phantom for self-calibration. Hence, the dose maps calculated based on SPECT images are quantitative and the dose value (Gy) is reliable as acquisition time, delay, scatter, and attenuation effects are considered. We extracted radiomics features from CT, SPECT, and dose maps. SPECT images were normalized to their own values from zero to 99 percentile of the image. A limited number of voxels get a very high intensity, recognized as outlier within the whole intensity distribution, due to the noisy nature of SPECT images. This is why we used 99 percentiles to keep the useful information while reducing noise.

Radiomic features can be extracted through two approaches, namely a fixed number of bins or fixed bin width. Selecting a higher bin width increases the focus on small changes in voxel values and heterogeneity, while at the same time, it increases the calculation time and the complexity of models and increases the effect of noise in the image on the decision made by the model. The optimum feature extraction parameter was decided both by considering the distribution of the data and the meaning of the voxel values. For example, for CT images, a voxel intensity less than 10 HUs can be due to changes in acquisition and reconstruction parameters. The same applies to SPECT and Dose map images.

The image preprocessing techniques, such as clipping that we used for feature extraction were empirical. We selected the bin width to balance the need to limit computational burdens and reduce noise while still capturing all the details and heterogeneities within the liver. We also used state-of-the-art FS methods and ML algorithms for classification tasks.

The optimal absorbed dose for HCC to achieve a therapeutic response varies depending on tumor's pathology and origin. Studies have reported a range of effective doses for achieving a response after SIRT, ranging from 91 to 490 Gy, depending on the type of microspheres used (resin or glass). Therefore, appropriate dosimetry must be tailored to the specific type of tumor [1, 34–37]. In the current study, since no cases experienced a complete response, our results indicated an ^90^Y-mean absorbed dose of approximately 245 Gy for partial response. However, this value should not be considered as a threshold for clinical use.

Regarding the identification of NGTDM coarseness as a distinguishing feature between NR and R groups, it is important to note that this feature measures the gray-level differences between the central pixel or voxel and its neighborhood, capturing the spatial rate of changes in gray-level intensities. Therefore, a ROI with more uniform gray levels, indicating a lower rate of spatial intensity changes, will have a high coarseness value [38]. However, as this is a preliminary study with a limited sample size, no firm conclusions can be drawn from these observations. Further evaluations are needed to confirm these results.

According to the BH statistical test results, none of the features showed statistically significant associations. We emphasize that a single parameter is not sufficient to decide about treatment response, since response to any treatment is multi-factorial (radiation sensitivity, individual characteristics of the patients, such as biological and physiological factors, age, and tumor type and microenvironment, etc.). This is why the use of AI is commended as it considers multiple information in decision making to stratify the patients and predict their outcomes.

Notably, the shape-based features calculated from WNL may be reliable since these structures are directly delineated on the CT images by our organ segmentation deep learning model [20]. However, the features derived from the manually delineated tumor structures on diagnostic CT or MRI following image registration may contain errors, as registration changes the shape of the structures. It should be noted that no single feature was evaluated regarding the response correlation, as we did not conduct any univariate analyses.

We developed multiple models using various available dataset prior to treatment (i.e. using ^99m^Tc dataset) and after ^90^Y treatment and extensively evaluated their potential in predicting the response and their limitations. The results demonstrated that ^90^Y-dosiomics modeling strategies outperformed the other models (AUC = 1). However, other promising results were also obtained using ^99m^Tc dosiomics (e.g., MAA-Dose-GLMB-RFE, AUC = 0.92) and radiomics (e.g., MAA-SPECT-CT-Clinical-MLP-MRMR, AUC = 0.91), as shown in Table 1. These findings indicate that it is possible to predict the response even before treatment, potentially providing invaluable insights for redirecting the treatment pathway. Given the extended time required to observe treatment response post-therapy, ^90^Y-predictive models can assist in identifying cases that may benefit from additional treatment cycles or a shift to alternative therapy modalities.

The results from ^99m^Tc-MAA DVH-based models did not demonstrate promising performance. In contrast, ^90^Y DVH-based models exhibited strong performance with AUCs consistently above 0.91. However, the performance of these promising models diminished when clinical features were added to DVH-driven features. Although we utilized state-of-the-art models, analysis and our study yielded encouraging results, we acknowledge several limitations. First, the limited sample size, collected in a single center, may have affected the robustness and generalizability of the models, and restricted us from performing external validation. However, we used strategies like bootstrapping and nested cross-validation to avoid overfitting. Moreover, the imbalance between the number of treatment non-responders and responders may introduce a bias in accuracy. To mitigate this bias, we applied the SMOTE technique. Nevertheless, additional training of these models with a more balanced patient cohort is necessary.

Given the retrospective nature of the study, we lacked access to important clinical features, such as Child–Pugh scores, which might be associated with treatment response. Another limitation is the short follow-up period, which can impact the mRECIST criteria results since the response (size reduction) may sometimes occur after more than three months post-therapy [39, 40]. Our focus on the feasibility of dosiomics and DVC metrics derived from challenging bremsstrahlung SPECT-based dosimetry for predicting tumor response meant that we did not explore different feature extraction parameterizations or selection approaches, which might influence feature values. Future studies may consider investigating this aspect. Furthermore, SPECT/CT is the most common and available imaging modality for SIRT. However, the poor spatial resolution and inherent noise of bremsstrahlung SPECT, along with challenges related to scatter correction, suggest that ^90^Y PET, and consequently generating high-resolution dose maps, may be more suitable for radiomics and dosiomics analysis.

Future investigation is necessary to tackle these limitations before we can draw firm conclusions regarding the efficacy of automated models for clinical use. Employing such models could ultimately assist clinicians in making reliable and informed decisions about patients and or treatment selection for patients with HCC.

## Conclusion

The findings and methodology of this study will help future studies to develop optimized SIRT treatment approaches in a prospective manner. Our study highlights the importance of considering features extracted not only from the tumor itself but also from OARs. Despite limitations, our research contributes to the growing body of evidence supporting the potential of automated models in improving treatment decision-making and patient outcomes in liver cancer management with SIRT.

## Supplementary Information

Below is the link to the electronic supplementary material.

Supplementary file1 (PDF 2191 KB)

## Data Availability

The data used in this work is not available.
