# Supplementary material for: Potential of Radiomics, Dosiomics, and Dose Volume Histograms for Tumor Response Prediction in Hepatocellular Carcinoma following 90Y-SIRT
Source: Mol Imaging Biol. 2025 Mar 10;27(2):201–14. doi: 10.1007/s11307-025-01992-8 (PMC12062168; doi:10.1007/s11307-025-01992-8)

Supplementary material

Introduction

Hepatocellular cancer is recognized as a significant cause of cancer-related mortality worldwide, with an increasing incidence rate over the past two decades [1]. Selective internal radiation therapy (SIRT) with ^{90}Y microspheres is an effective treatment for highly burdened liver cancer. ^{99m}Tc macroaggregated albumin (^{99m}Tc-MAA) is utilized as a theranostic counterpart to simulate ^{90}Y distribution and shunt study. In the context of personalized patient management, treatment planning and pre-therapy dosimetry are performed based on the ^{99m}Tc-MAA SPECT/CT scans with the goal of maximizing the tumor absorbed dose and limiting unwanted damage to the surrounding normal tissues [2].

Post-treatment verification and dosimetry involve the acquisition of ^{90}Y SPECT/CT or PET/CT scans. Currently, one of the most accurate dosimetry methods is voxel-level dosimetry, which accounts for heterogeneity of tumor and dose distribution by calculating absorbed dose values within each voxel [3]. Studies have indicated that higher treatment radiation doses and ^{90}Y injected activity can improve overall treatment response, only if organs at risk (OARs) are not overdosed [4-6].

Utilizing prospective treatment planning and post-treatment verification through voxel-level dosimetry can improve tumor response, prolong overall survival, and predict treatment outcomes [7-9]. These data can also be valuable for informing future treatments and establishing a dose-effect relationship, addressing a key challenge in internal dosimetry.

Dose-volume histograms (DVHs) are reliable tools that can be used to evaluate the absorbed dose received by any specific volume. DVHs provide a quantitative summary of dose distribution through visually depiction and facilitate the comparison of two different dose distributions. However, one of the limitations of DVHs is that they lack spatial information and are not able to locate the regions where dose inhomogeneities occur [10, 11].

Moreover, tumors are also composed of heterogenous sub-regions at their genetic and histological levels, which exist due to variations in angiogenesis, cellularity, necrosis, etc. There are indications that tumor resistance or treatment failure is associated with tumor heterogeneity [12-14].

Materials and Methods

Feature selection and machine learning modeling

To train the models, we employed a 3-fold nested cross-validation (CV) approach, comprising an inner and an outer CV loop, to prevent overfitting and ensure a more reliable performance. Features extracted from training sets of each strategy were normalized to their Z-score, with the resulting mean and standard deviation applied to corresponding features extracted from the test datasets within the outer loop.

Different machine learning (ML) algorithms, combined with different feature selection (FS) methods aimed at identifying the most relevant features and eliminating redundant ones, were

utilized. ML modeling was carried out using eight different algorithms, including Decision Tree (DT), Generalized Linear Mixed Model Boosting (GLMB), Logistic Regression (LR), Multiple Layer Perceptron (MLP), Naïve Bayes (NB), Random Forest (RF), Support Vector Machine (SVM), and Extreme Gradient Boosting (XGB). We used five different FS methods including ANOVA, Kruskal, Minimum Redundancy Maximum Relevant (MRMR), Randomized Ensemble Feature Importance (Relief), and Recursive Feature Elimination (RFE). The redundant features were removed using the Spearman's rank correlation coefficient. A rho of 0.90 was used as threshold.

Hyperparameter optimization was carried out using Grid Search with 3-fold cross-validation within the inner CV loop, and the best values employed for model training. Given the small sample size, we also generated 1000 bootstrap samples with replacement for ROC curves. The dataset was imbalanced between the number of treatment non-responders and responders. Synthetic Minority Oversampling Technique (SMOTE) was used on the training sets to overcome any biases in model performance due to unbalanced dataset. SMOTE was used during hyperparameter optimization on the inner training dataset, and once the best hyperparameters were chosen, it was also applied to the outer training dataset. The final trained model was evaluated on the outer test dataset. Eventually, we ended up trying 1440 models (resulting from 8 ML× 5 FS ×6 categories × 6 subcategories). Supplemental Table 1 summarizes the hyperparameters and their ranges for each classifier.

For every model, a confusion matrix was computed, detailing true negative (TN), true positive (TP), false negative (FN), and false positive (FP) rates. Model performance was assessed using metrics, such as Area Under the Receiver Operating Characteristic Curve (AUC), Accuracy (ACC), sensitivity (SEN), and specificity (SPE). To ascertain the most robust models, Delong statistical test was employed to compare AUC values, with a P-value < 0.05 indicating statistical significance.

Discussion

^{90}Y SIRT is a well-established and generally well-tolerated treatment option for patients with unresectable liver cancer. However, early prediction of treatment response is crucial for optimizing patient outcomes. Leveraging artificial intelligence as a tool in this context can provide valuable insights and aid in the decision-making process [15].

The primary objective of our study was to assess the feasibility and potential value of radiomic and dosiomic features, as well as DVC values, in predicting treatment response to SIRT. To achieve this, we evaluated multiple machine-learning models incorporating high-dimensional radiomic and dosiomic features, as well as DVC-derived factors extracted from pre- and post-therapy images and dose maps.

References

1. Sung H, Ferlay J, Siegel RL, Laversanne M, Soerjomataram I, Jemal A, et al. Global cancer statistics 2020: GLOBOCAN estimates of incidence and mortality worldwide for 36 cancers in 185 countries. CA: a cancer journal for clinicians. 2021;71:209-49.
2. Garin E, Lenoir L, Rolland Y, Edeline J, Mesbah H, Laffont S, et al. Dosimetry based on 99mTc-macroaggregated albumin SPECT/CT accurately predicts tumor response and survival in

hepatocellular carcinoma patients treated with 90Y-loaded glass microspheres: preliminary results. Journal of Nuclear Medicine. 2012;53:255-63.

3. Xiao Y, Roncali E, Hobbs R, St James S, Bednarz B, Benedict S, et al. Toward individualized voxel-level dosimetry for radiopharmaceutical therapy. International journal of radiation oncology, biology, physics. 2021;109:902-04.
4. Chansanti O, Jahangiri Y, Matsui Y, Adachi A, Geeratikun Y, Kaufman JA, et al. Tumor dose response in yttrium-90 resin microsphere embolization for neuroendocrine liver metastases: a tumor-specific analysis with dose estimation using SPECT-CT. Journal of Vascular and Interventional Radiology. 2017;28:1528-35.
5. Eaton BR, Kim HS, Schreibmann E, Schuster DM, Galt JR, Barron B, et al. Quantitative dosimetry for yttrium-90 radionuclide therapy: tumor dose predicts fluorodeoxyglucose positron emission tomography response in hepatic metastatic melanoma. Journal of Vascular and Interventional Radiology. 2014;25:288-95.
6. Kao Y-H, Steinberg JD, Tay Y-S, Lim GK, Yan J, Townsend DW, et al. Post-radioembolization yttrium-90 PET/CT-part 2: dose-response and tumor predictive dosimetry for resin microspheres. EJNMMI research. 2013;3:1-12.
7. Cheng B, Villalobos A, Sethi I, Wagstaff W, Galt J, Brandon D, et al. Determination of tumor dose response thresholds in patients with chemorefractory intrahepatic cholangiocarcinoma treated with resin and glass-based Y90 radioembolization. CardioVascular and Interventional Radiology. 2021;44:1194-203.
8. Garin E, Tselikas L, Guiu B, Chalaye J, Edeline J, de Baere T, et al. Personalised versus standard dosimetry approach of selective internal radiation therapy in patients with locally advanced hepatocellular carcinoma (DOSISPHERE-01): a randomised, multicentre, open-label phase 2 trial. The lancet Gastroenterology & hepatology. 2021;6:17-29.
9. Dewaraja YK, Devasia T, Kaza RK, Mikell JK, Owen D, Roberson PL, et al. Prediction of tumor control in 90Y radioembolization by logit models with PET/CT-based dose metrics. Journal of Nuclear Medicine. 2020;61:104-11.
10. Datta NR, Das KJ, Balasubramanium R, Ayyagari S. Spatial information on dose distribution using multisectional dose-volume histograms. Med Dosim. 1996;21:19-22.
11. Mansouri Z, Salimi Y, Akhavanallaf A, Shiri I, Teixeira E, Hou X, et al. Deep transformer-based personalized dosimetry from SPECT/CT images: A hybrid approach for [177Lu]Lu-DOTATATE radiopharmaceutical therapy. Eur J Nucl Med Mol Imaging. 2024;51:1516-29.
12. Lambin P, Leijenaar RT, Deist TM, Peerlings J, De Jong EE, Van Timmeren J, et al. Radiomics: the bridge between medical imaging and personalized medicine. Nature reviews Clinical oncology. 2017;14:749-62.
13. Abdollahi H, Chin E, Clark H, Hyde DE, Thomas S, Wu J, et al. Radiomics-guided radiation therapy: opportunities and challenges. Physics in Medicine & Biology. 2022;67:12TR02.
14. Abdollahi H. Radiotherapy dose painting by circadian rhythm based radiomics. Medical Hypotheses. 2019;133:109415.
15. Arabi H, AkhavanAllaf A, Sanaat A, Shiri I, Zaidi H. The promise of artificial intelligence and deep learning in PET and SPECT imaging. Phys Med. 2021;83:122-37.

Table 1. Patient characteristics and values of laboratory tests are reported as median ± SD. * ECOG: Eastern Cooperative Oncology Group. The p-values for continuous and categorical variables are reported based on the Mann-Whitney U test and Fisher's Exact statistical tests, respectively.

CHARACTERISTICS	*RESPONDERS N=5*	*NON-RESPONDERS N=12*	*P-VALUE*
**SEX**	5 Males	11 males, 1 Female	Fisher's p>0.05
**AGE**	Median:77±19.64 [range; 39-84yrs]	Median:71.5±7.8 [range; 53-81yrs]	p>0.05
**ETIOLOGY**			
CHRONIC HEPATITIS	-	1	
VIRAL	1	3	Fisher's
CIRRHOTIC + ETOH(ALCOHOL)	1	3	
CIRRHOSIS	1	2	p>0.05
NONCIRRHOTIC	2	3	
**CHILD PUGH**			
A	2	3	Fisher's
B	1	1	p>0.05
UNKNOWN	2	8	
**PREVIOUS TREATMENT**			
NON	3	9	Fisher's
CHEMOEMBOLIZATION	1	1	p>0.05
CHEMOEMBOLIZATION+ BÉVACIZUMAB	-	1	
CHEMOEMBOLIZATION + SORAFENIB	1	-	
PREVIOUS SIRT (4 YEARS AGO)	-	1	
**EXTRAHEPATIC METASTASIS**	No	Yes: No (1: 11)	Fisher's p>0.05
**PORTAL VEIN THROMBOSIS (PVT)**	Yes: No (2:3)	Yes: No (3:9)	Fisher's p>0.05
**HEPATITIS**	Yes: No (4:1) (B:2, C:1, D:1)	Yes: No (3:9)	Fisher's p>0.05
**ASCITES**	Yes: No (1:4)	Yes: No (1:11)	Fisher's p>0.05
**LIVER CIRRHOSIS**	Yes: No (3:2)	Yes: No (9:3)	Fisher's p>0.05
**AIM OF SIRT**			
LOBECTOMY (RIGHT: LEFT)	4 (3 :1)	8(7: 1)	Fisher's
PALLIATIVE	0	1	p>0.05
SEGMENTECTOMY	1	3	
**ALPHA FETOPROTEIN (AFP) MEAN ± SD (μG/L)**	19.5 ± 532.4	242.5 ± 23313.32	p>0.05
**ALBUMIN MEAN ± SD (G/L)**	34 ± 6.7	40.5 ±3.6	p>0.05
**TOTAL BILIRUBIN (μMOL/L)**	14 ± 22.95	11 ± 33.9	p>0.05
**ASPARTATE AMINOTRANSFERASE (AST) (U/L)**	55 ± 13.3	37 ± 71.6	p>0.05
**ALANINE AMINOTRANSFERASE (ALT) (U/L)**	51 ± 11.3	40.5 ± 74.47	p>0.05
**HEMOGLOBIN (G/L)**	133 ± 26.9	137.5 ± 20.98	p>0.05
**LEUCOCYTE COUNT (×10^9/L)**	4.7 ± 1.6	6.15 ± 1.38	p>0.05
**PLATELET COUNT (×10^9/L)**	138 ± 63.12	148±88.06	p>0.05
**PERFORMANCE (ECOG* SCALE)**	0:1 (4:1)	0:1 (8:4)	p>0.05
**BASELINE TUMOR VOLUME (ML)**	205.03 ± 118.72	159.76 ± 243.4	p>0.05

Table 2. The hyperparameters and their ranges used for training of each classifier.

Classifier	Hyper parameter	Range

XGB	eta	0.025, 0.05, 0.1, 0.3
	max_depth	2-10, step=1
	nrounds	50-1000, step=50
	colsample_bytree	0.4, 0.6, 0.8, 1.0
	subsample	0.5, 0.75, 1.0
	gamma	0, 0.05, 0.1, 0.5, 0.7, 0.9, 1.0
	min_child_weight	1, 2, 3
SVM	cost	0.1-10, step=0.1
	gamma	0.1-10, step=0.1
DT	minsplit	5-20, step=1
	minbucket	3-10, step=1
RF	ntree	50-1000, step=50
	mtry	1-10, step=1
	nodesize	1-20, step=1
MLP	sizee	1-10, step=1
GLMB	mstop	50, 500, step=500
LR	-	-
NB	-	-

Table 2. The mean ± SD of the dose metrics derived from 99mTc-MAA and 90Y DVH and BVHs along with the p-values from Mann-Whitney statistical test.

	Dose Metrics	*TL*			*NPL*			*WNL*		
		R	NR	P-value	R	NR	P-value	R	NR	P-value
^{99m}Tc-MAA	Volume (ml)	260 ± 103.8	270.2 ± 334.5	>0.05	851.8 ± 510.5	945.5±511.2	>0.05	1223.6 ± 344.6	1638.2±354.2	0.03
	Dmax (Gy)	972.3 ± 188.4	1437.4±898.02	>0.05	494.2 ± 135.9	703.7±416.1	>0.05	511.4 ± 142.3	702.4±417.4	>0.05
	Dmean (Gy)	341.2±121.7	382.4±202.5	>0.05	66.1± 67.1	83.5±78.6	>0.05	33±14.5	41.6±25.9	>0.05
	Dmin (Gy)	29.1 ± 20.38	47.3±31.2	>0.05	1.3±1.5	3.9±9.2	>0.05	0.22±0.17	0.26±0.23	>0.05
	D50 (Gy)	30.9.7± 125.4	324.4±166.1	>0.05	50.6±68.9	57.09±65.9	>0.05	11.01 ± 7.2	16.5±27.2	>0.05
	D70 (Gy)	223.5±97.9	240.1±102.8	>0.05	30.5±45.8	35.07±49.1	>0.05	4.2±2.7	7.6±11.1	>0.05
	D95 (Gy)	110.3 ± 59.85	131.9±65.8	>0.05	9.3 ± 14.8	12.5±21.9	>0.05	1.3± 0.9	1.4±1.1	>0.05
	D98(Gy)	77.7± 42.3	102.1±59.8	>0.05	5.8 ± 8.8	9.05±16.9	>0.05	0.97 ± 0.6	1.05±0.8	>0.05
	HI	731.8 ± 316.3	1090.8±1426.4	>0.05	_	_	_	_	_	_
	V20(ml)	_____	_____	____	451.4 ± 309.3	572.08±468.8	>0.05	447.3 ± 272.4	589.6±445.6	>0.05
	V20(%)	_	_	_	57.5 ± 27.6	93.4±114.8	>0.05	34.5± 15.6	66.7±115.7	>0.05
	V30 (ml)	_	_	_	359.4 ± 241.6	461.2±460.7	>0.05	355.4 ± 217.2	467.5±449.5	>0.05
	V30(%)	_	_	_	48.4 ± 30.06	83.6±110.6	>0.05	27.7 ± 13.2	59.3±110.8	>0.05
	V50(ml)	_		_	254.7 ± 164.3	362.8±416.3	>0.05	250.9±150.3	364.1±409.7	>0.05
	V50(%)	_	_	_	38.05 ± 31.8	42.05±30.6	>0.05	19.9 ±10.05	22.34±17.1	>0.05
	V70 (ml)	_	_	_	195.09 ± 123.6	296.1±370.9	>0.05	191.78 ± 115.09	296.6±365.8	>0.05
	V70(%)	_	_	_	31.5 ± 31.2	60.5±92.8	>0.05	15.4 ± 8.2	43.1±92	>0.05
	V90(ml)	__	_	_	146.09 ± 90.5	247.7±333.9	>0.05	144.09 ± 85.6	247.6±328.9	>0.05
	V90(%)	_	_	_	26±30.12	51.9±82.2	>0.05	11.7±6.5	37.06±81.06	>0.05
	V120(ml)	229.3±94.4	177.46±112.8	>0.05	89.5±56.1	189.8±265.8	>0.05	88.4±54.5	189.5±262.5	>0.05
	V120(%)	88.41±54.5	88.9±21.5	>0.05	19.1±27.2	40.1±63.5	>0.05	7.3±4.9	28.3±62.1	>0.05
	V205(ml)	178.9±99.4	143.4±76.4	>0.05	28.6±26.4	66.2±67.5	>0.05	28.1±26.2	66.3±69.06	>0.05
	V205(%)	68.8±20.4	74.3±23.4	>0.05	9.1±16.9	19.6±36.6	>0.05	2.5 ± 3.09	13.8±34.4	>0.05
	V400(ml)	85.2±91.8	60.2±32.2	>0.05	1.5±2	11.07±15.9	>0.05	1.6±2	11.5±18.4	>0.05
	V400(%)	32.5±23.9	29.1±23	>0.05	0.7±1.4	3.4±7.07	>0.05	0.16±0.2	1.7±3.8	>0.05
	BED-max (Gy)	3195.6±1033.7	6266.1±9185.7	>0.05	1465.4±626.3	2832.4±3226.2	>0.05	1552.5±652.6	2858.1±3209.6	>0.05
	BED-mean(Gy)	709.8± 356.3	793.7±589.4	>0.05	104.6±124.05	156.2±213.2	>0.05	49.3±25.03	72.1±51.4	>0.05
	BED-min(gy)	31.8±23.1	53.4±37.7	>0.05	0	0.004±0.01	>0.05	0.23±0.17	0.26±0.23	>0.05
	BED-D50(Gy)	557.5±326.6	545.5±350.04	>0.05	80.7±129.6	90.94±132.1	>0.05	11.6±7.8	20.05±37.7	>0.05
	BED-D70(Gy)	355.04±212.7	360.03±164.03	>0.05	45.9±78.7	52.07±87.09	>0.05	4.3±2.8	8.22±12.9	>0.05
	BED-D95(Gy)	144.6±91.6	172.8±97.8	>0.05	14.2±25.4	17.4±32.3	>0.05	1.3±0.9	1.4±1.1	>0.05
	BED-D98(Gy)	94.7±57.1	129.5±87.8	>0.05	8.7±14.8	12.4±24.4	>0.05	0.95±0.65	1.02±0.8	>0.05
	BED_HI	1491.3±667.2	2466.2±3235.5	>0.05	_	_	_	_	_	_
	BED-V20(ml)	_	_	_	431.8±297.9	546.8±449.6	>0.05	462.1±280.8	604.8±448.6	>0.05
	BED-V20(%)	_	_	_	53.2±23	56.7±23.7	>0.05	35.6±16	35.2±19.9	>0.05
	BED-V30(ml)	_	_	_	355.7 ±241.7	477.3±429.2	>0.05	377.1±230.04	517.4±435.6	>0.05
	BED-V30(%)	_	_	_	46.1 ± 25.26	49.8±26.4	>0.05	29.3±13.7	29.7±19.01	>0.05
	BED-V50(ml)	_	_	_	264.8 ± 174.5	392.9±401.5	>0.05	278.6±168.1	420.1±410.5	>0.05
	BED-V50(%)	_	_	_	37.4 ± 27.6	41.9±28.1	>0.05	21.9±10.9	23.7±17.58	>0.05
	BED-V70(ml)	_	_	_	216.3 ± 139.2	339.7±374.9	>0.05	226.4±135.4	361.03±382.8	>0.05
	BED-V70(%)	_	_	_	32.6 ± 28.2	36.9±27.8	>0.05	18.04±9.36	20.3±16.5	>0.05
	BED-V90(ml)	_	_	__	181.4 ± 115.4	299.5±348.8	>0.05	189.1±113.4	317.03±355.8	>0.05
	BED-V90(%)	_	_	_	28.9 ± 28.1	32.9±26.8	>0.05	15.2±8.1	17.7±15.4	>0.05
	BED-V120(ml)	239.6 ± 94.3	185.2 ± 124.1	>0.05	138.7 ± 86.8	254.3±318.9	>0.05	144.5±85.9	267.9±323.6	>0.05
	BED-V120(%)	92.8 ± 7.01	90.9 ± 20.7	>0.05	24.2 ± 27.4	28.2±25.2	>0.05	11.7±6.5	14.9±14.2	>0.05
	BED-V205(ml)	210.4 ± 96.2	165.3 ± 98.9	>0.05	66.9 ± 43.7	164.8±212.9	>0.05	66.9±43.7	173.3±215.8	>0.05
	BED-V205(%)	80.9 ± 14.2	84.6 ±22.1	>0.05	15.5 ± 23.6	19.1±20.6	>0.05	5.9±4.5	9.7±9.7	>0.05
	BED-V400(ml)	149.5 ± 102.08	113.5 ± 63.2	>0.05	22.2 ± 21.5	58.02±55	>0.05	23.1±22.6	62.14±59.4	>0.05
	BED-V400(%)	57.6 ± 24.2	58.9 ± 24.1	>0.05	7.3 ± 13.6	8.5±13	>0.05	2.1±2.6	3.9±3.8	>0.05
^{90}Y	Dmax (Gy)	536.2 ± 136	1055.3±1128.7	>0.05	302.4 ± 98.4	453.7±333.1	>0.05	280.8±111.2	453.7±333.1	>0.05
	Dmean (Gy)	244.4 ± 83.3	312.1±161.3	>0.05	79.5 ± 45.7	69.8±33.8	>0.05	49.7±25.6	44.8±23.2	>0.05
	Dmin (Gy)	64.9 ± 32.5	56.7±35.8	>0.05	12.5 ± 10.5	8.9±5.7	>0.05	3.09±1.5	3.08±1.9	>0.05
	D50 (Gy)	229.1 ± 76.9	263.6±114.7	>0.05	67.4 ± 42.8	52.7±32.2	>0.05	33±20.9	29.4±23.8	>0.05
	D70 (Gy)	187.4 ± 69.4	186.7±94	>0.05	46.6 ± 35.7	38.5±24.8	>0.05	18.3±11.4	19.07±15.2	>0.05
	D95 (Gy)	121.9 ± 57.7	112.6±64.8	>0.05	25.5 ± 23.1	20.2±11.6	>0.05	8.3±5.4	8.1±5.7	>0.05
	D98(Gy)	107.2 ± 53.8	95±55.3	>0.05	20.2 ±17.3	16.5±9.3	>0.05	6.6±4.4	6.4±4.34	>0.05
	HI	364.6 ±106.6	742.9±401.2	>0.05	_	_	_	_	_	___
	V20(ml)	_	_	_	672.2±421.4	786.9±456.3	>0.05	764.8±373.05	1010.1±470.9	>0.05
	V20(%)	_	_	_	84.1±18.05	82.8±16.8	>0.05	60.5±25.2	58.02±21.6	>0.05
	V30 (ml)	_	_	_	562.03±366.9	625.9±453.06	>0.05	611.7±341.5	745.05±200.1	>0.05
	V30(%)	_	_	_	72.06±25.6	66.7±25.8	>0.05	48±23.7	41.9±22.08	>0.05
	V50(ml)	_	_	_	415.7±283.6	435.8±431.8	>0.05	432.5±267	481.1±462.4	>0.05
	V50(%)	_	_	_	56.6±31.3	47.1±29.9	>0.05	33.9±19.4	26.2±19.8	>0.05
	V70 (ml)	_	_	_	308.3±218.2	328.5±397.1	>0.05	315.7±210.06	349.2±414.7	>0.05
	V70(%)	_	_	_	45.2±32.5	34.7±26.9	>0.05	24.9±16.02	18.5±17.5	>0.05
	V90(ml)	_	_	_	222.8±172.4	252.5±342.5	>0.05	226.6±169.1	262.1±350.9	>0.05
	V90(%)	_	_	_	35±30.5	26.06±22.7	>0.05	18.07±13.3	13.7±14.8	>0.05
	V120(ml)	230.8±55.1	193.2±124.6	>0.05	133.02±130.3	160.8±215.8	>0.05	132.9 ± 126.4	163.4±215.7	>0.05
	V120(%)	88.6±9.6	81.4±22.4	>0.05	22.6±23.6	16.7±15.4	>0.05	10.7±10.1	8.6±9.4	>0.05
	V205(ml)	135±84.1	137.5±73.4	>0.05	26±42.6	36.3±40.5	>0.05	25.8±42.7	36.6±40.5	>0.05
	V205(%)	53.6±31.6	26.4±62.4	>0.05	5.5±7.6	4.4±5.6	>0.05	2.2±3.5	2.1±2.5	>0.05
	V400(ml)	29.1±42.1	44.3±46.2	>0.05	0.08±0.19	5.6±9.9	>0.05	0.08±0.19	5.7±10.1	>0.05
	V400(%)	11.5±14.8	21.4±21.3	>0.05	0.05±0.12	0.5±0.7	>0.05	0.01±0.02	0.3±0.6	>0.05
	BED-max (Gy)	1226.4±454.8	6368.8±11393.4	>0.05	613.4±360	1606.8±2291.7	>0.05	613.4±360	1606.8±2291.7	>0.05
	BED-mean(Gy)	413.3±194.4	725.5±671.3	>0.05	113.4±81.3	100.2±56.7	>0.05	69.7±41.4	65.1±40.5	>0.05
	BED-min(gy)	76.5±42.9	66.7±46.5	>0.05	0	0	>0.05	3.1±1.5	3.1±1.9	>0.05
	BED-D50(Gy)	359.8±167.4	449.9±256.8	>0.05	92.07±71.1	69.8±50.1	>0.05	38.4±27.01	34.6±33.3	>0.05
	BED-D70(Gy)	276.4±138.7	284.8±177.07	>0.05	60.13±54.6	49.1±36.6	>0.05	20±13.2	21.2±18.9	>0.05
	BED-D95(Gy)	162.04±96.8	150.3±100.8	>0.05	30.7±31.6	24.6±15.5	>0.05	8.6±5.8	8.5±6.2	>0.05
	BED-D98(Gy)	138.7±86.3	121.9±80.8	>0.05	25.6±26.4	20.1±12.1	>0.05	6.9±4.7	6.6±4.5	>0.05
	BED_HI	563.7±208.1	1698.9±1273.1	>0.05	_	_	___	_	_	___
	BED-V20(ml)	_	_	_	644.03±411.4	758.2±429.5	>0.05	790.8±377.8	1056.1±462.1	>0.05
	BED-V20(%)	_	_	_	79.8±17.5	79.02±12.8	>0.05	62.6±25.3	60.7±21.3	>0.05
	BED-V30(ml)	_	_	_	554.03±365.7	624.6±425.6	>0.05	648.07±351	804.9±498.3	>0.05
	BED-V30(%)	_	_	_	70.02±23.7	65.6±20.6	>0.05	50.9±24.3	45.6±22.1	>0.05
	BED-V50(ml)	_	_	_	432.5±294.4	460.1±423.1	>0.05	481.8±289.1	548.5±480.5	>0.05
	BED-V50(%)	_	_	_	57.3±28.5	49±27.4	>0.05	37.7±20.5	30.1±20.7	>0.05
	BED-V70(ml)	_	_	_	350.8±243.6	370.01±403.4	>0.05	383.4±244.1	422.5±444.04	>0.05
	BED-V70(%)	_	_	_	49.07±30.7	39.3±27.3	>0.05	30.1±18.13	22.8±18.9	>0.05
	BED-V90(ml)	_	_	_	286.5±204.08	309.5±381.6	>0.05	310.1±207.5	343.7±411.4	>0.05
	BED-V90(%)	_	_	_	42.08±31.01	32.3±25.3	>0.05	24.5±15.8	18.2±17.4	>0.05
	BED-V120(ml)	247.5±60.07	209.8±151.2	>0.05	211.8±162.6	243.7±336.4	>0.05	227.2±169.4	262.8±351.6	>0.05

BED-V120(%)	94.6±5.2	85.3±19.6	>0.05	33.3±29.5	24.8±21.8	>0.05	18.1±13.3	13.7±14.9	>0.05
BED-V205(ml)	196.3±58.04	170..7±96.6	>0.05	94.7±104.6	116.3±145.2	>0.05	99.1±108.5	121.4±146.2	>0.05
BED-V205(%)	76.1±18.9	74.9±24.8	>0.05	17.01±19.5	12.3±11.2	>0.05	8.1±8.7	6.5±6.6	>0.05
BED-V400(ml)	97.1±86.7	105.6±57.2	>0.05	18.2±29.9	28.8±34.4	>0.05	18.7±30.8	30±35.6	>0.05
BED-V400(%)	38.7±31.6	49.5±25.9	>0.05	4.1±6.1	3.5±4.5	>0.05	1.6±2.5	1.8±2.2	>0.05

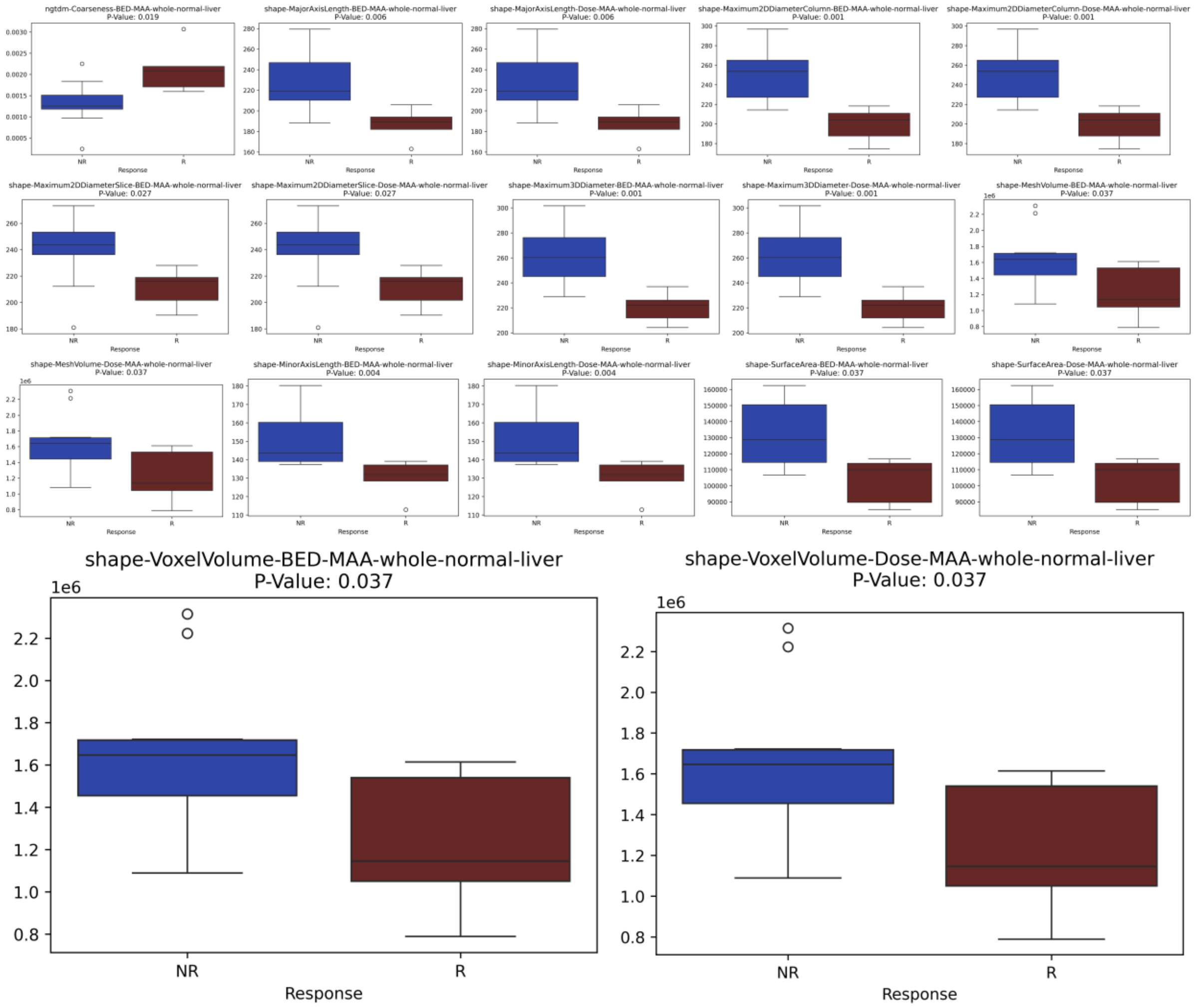

Figure 1. The statistically significant different MAA-Dosiomic features between R and NR groups, along with their distribution in box plots.

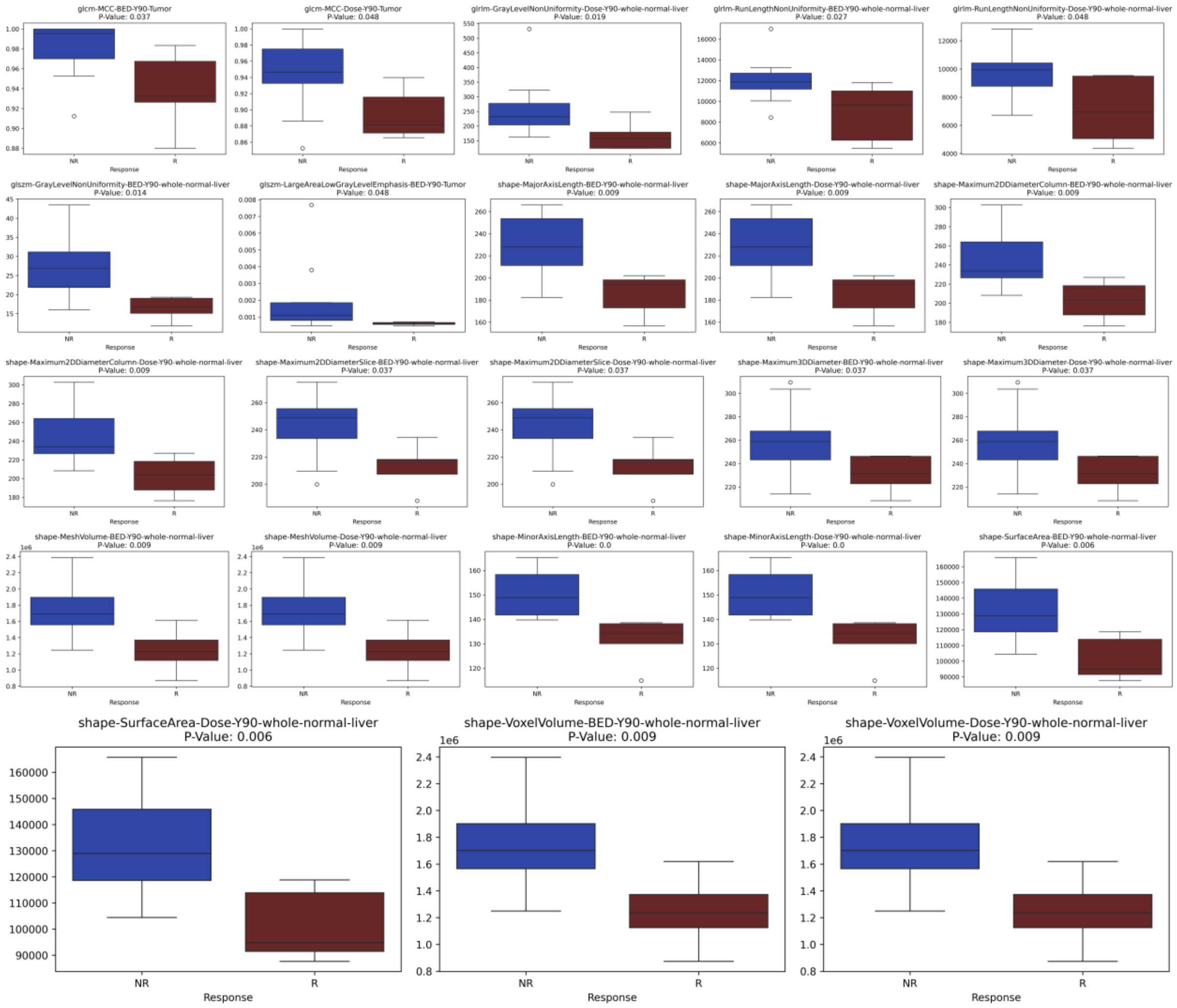

Figure 2. The statistically significant different ^{90}Y-Dosiomic features between R and NR groups, along with their distribution in box plots.

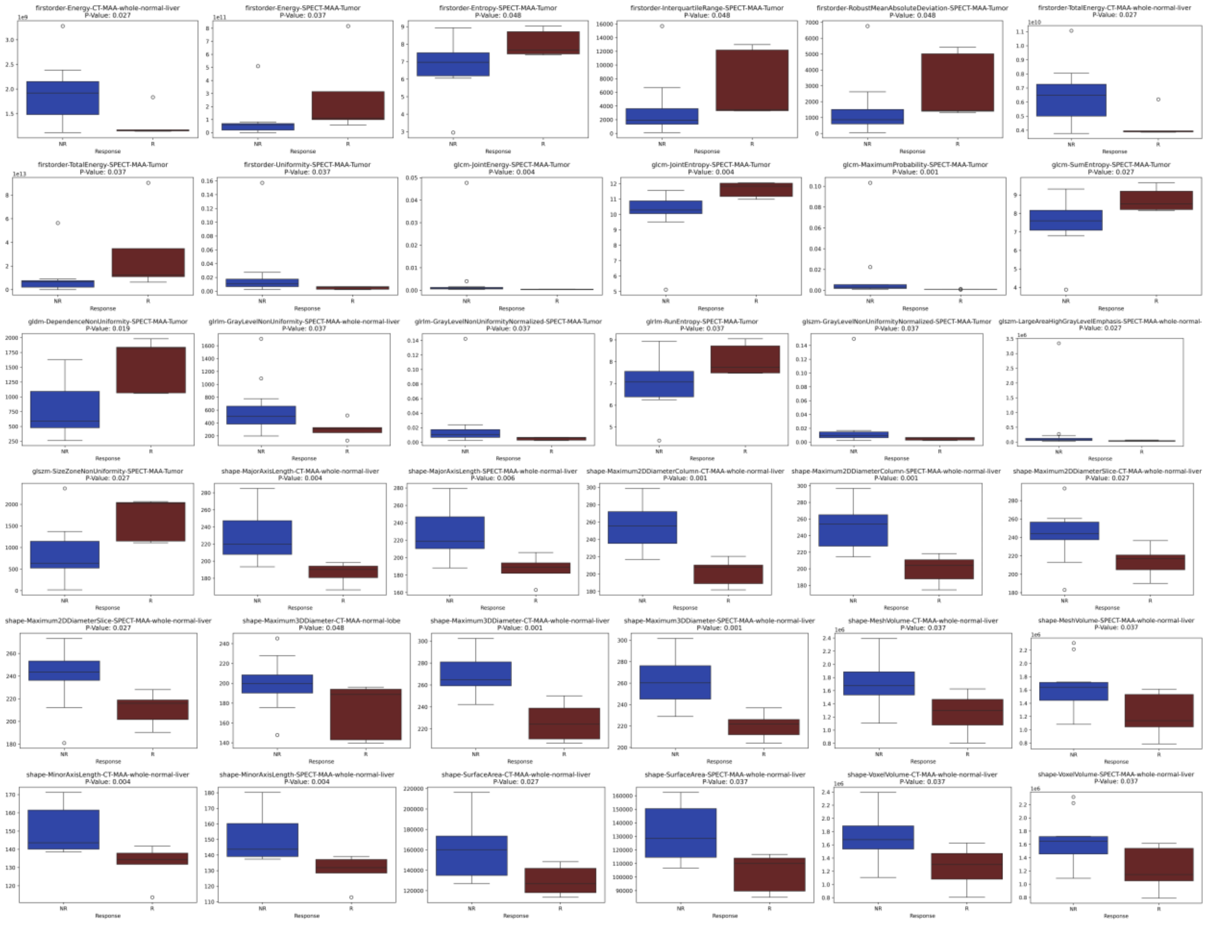

Figure 3. The statistically significant different MAA-Radiomic features between R and NR groups, along with their distribution in box plots.

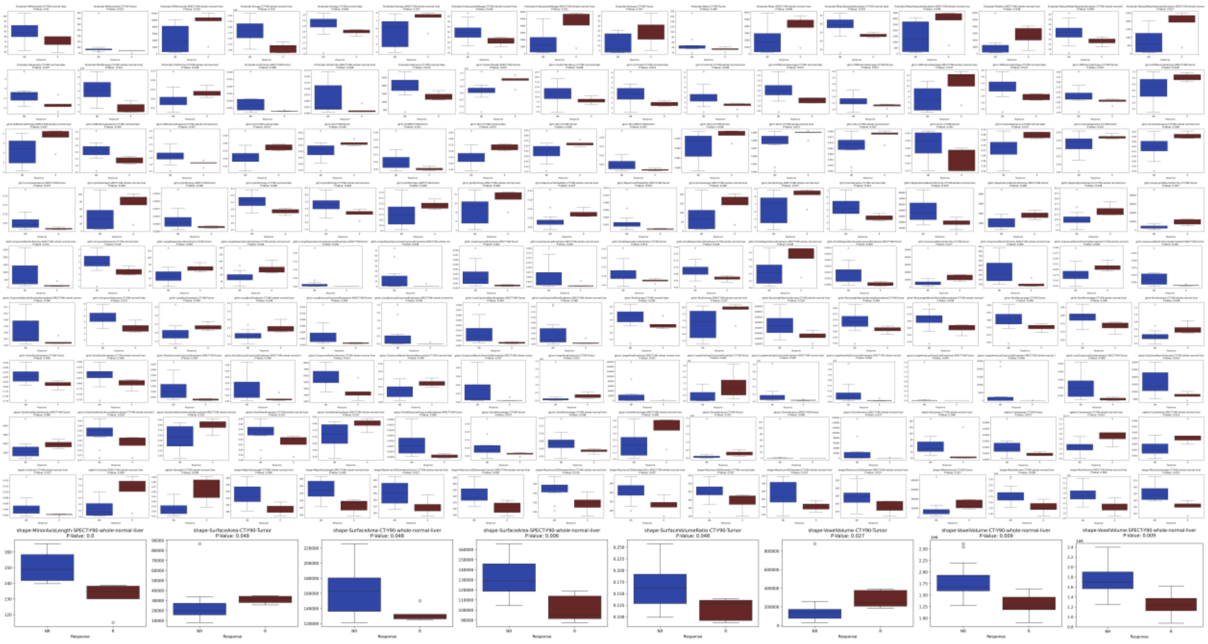

Figure 4. The statistically significant different ^{90}Y-Radiomic features between R and NR groups, along with their distribution in box plots.

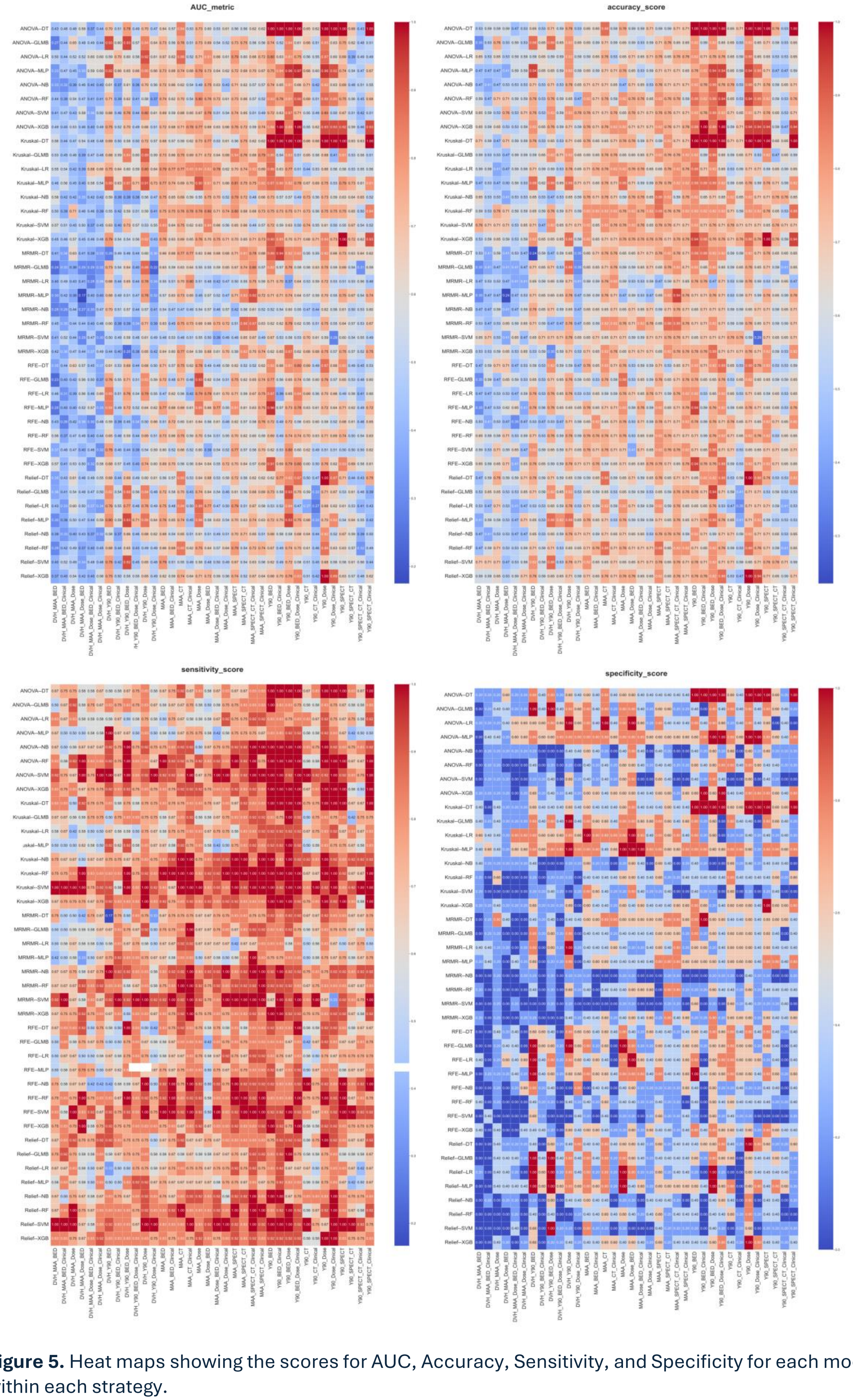

Figure 5. Heat maps showing the scores for AUC, Accuracy, Sensitivity, and Specificity for each model within each strategy.

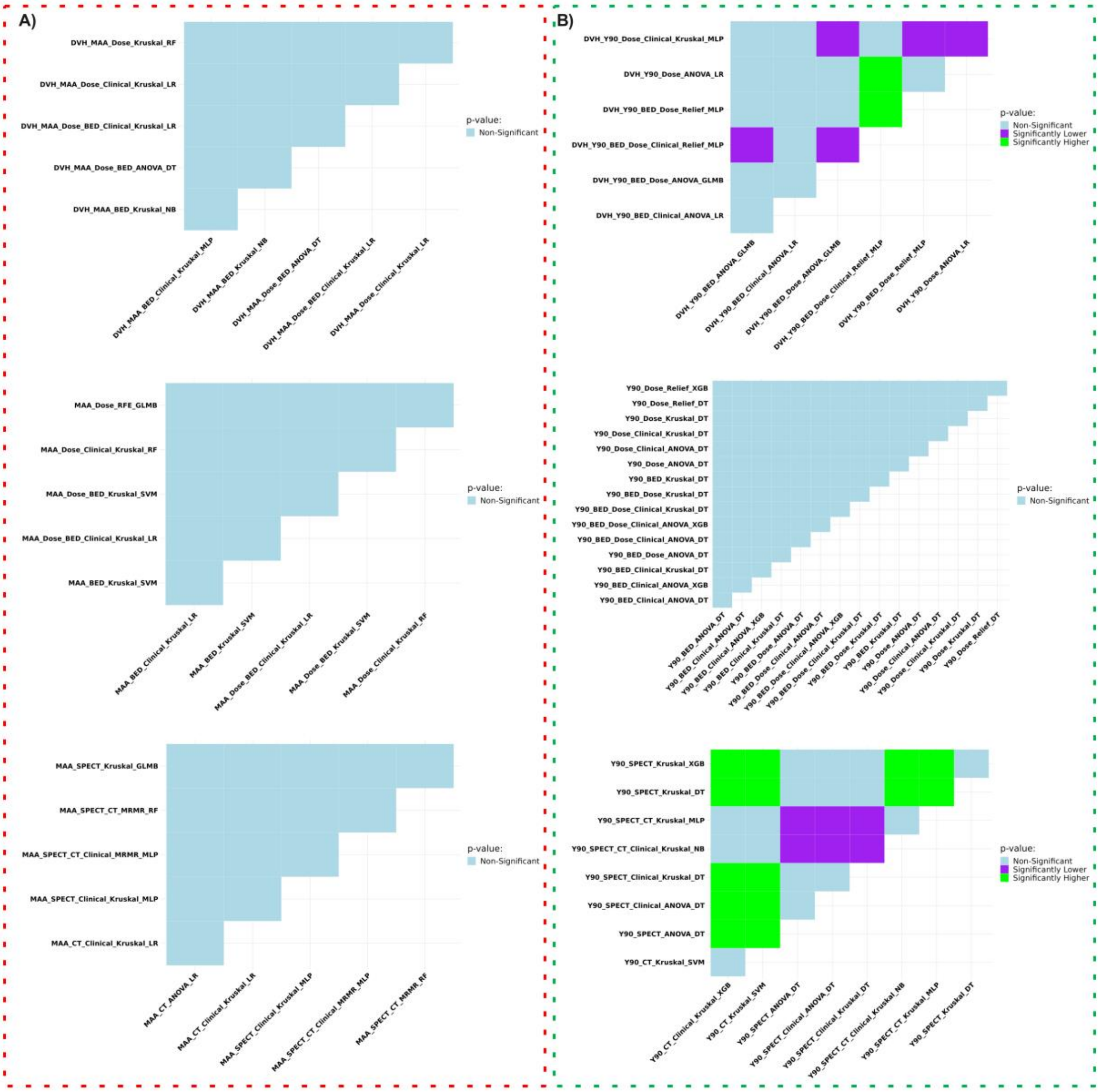

Figure 6. The visualization includes p-values from Delong statistical tests comparing well-performing models within each strategy A) for MAA models B) for ^{90}Y models. Blue squares indicate statistically non-significant correlations between the models, while purple and green squares show significantly lower and higher model performance, respectively.

Table 3. The selected features used for model training in each fold for high-performance models.

		FOLD-1	FOLD-2	FOLD-3
^{99M}TC-MAA	DVH-Dose-Kruskal	MAA-Dose-MAX-TL-Gy<br>Volume-TL-ml<br>Volume-WNL-ml<br>MAA-Dose-V205(%)-WNL<br>LSF	Volume-WNL-ml<br>Volume-NPL-ml<br>MAA-Dose-D98-TL-Gy<br>MAA-Dose-V400(ml)-TL<br>MAA-Dose-D50-WNL-Gy	Volume-WNL-ml<br>LSF<br>MAA-TNR-WNL<br>MAA-Dose-V50(%) -WNL<br>MAA-Dose-V205(ml)-TL
	Dosiomics MAA-Dose-RFE	glszm-LargeAreaHighGrayLevelEmphasis-Dose-MAA-NPL<br>gldm-LowGrayLevelEmphasis-Dose-MAA-Tumor<br>shape-Maximum3DDiameter-Dose-MAA-WNL<br>shape-Maximum2DDiameterColumn-Dose-MAA-WNL<br>glszm-ZoneEntropy-Doses-MAA-WNL	shape-MinorAxisLength-Dose-MAA-WNL<br>shape-MinorAxisLength-Dose-MAA-NPL<br>shape-MajorAxisLength-Dose-MAA-WNL	shape-Flatness-Dose-MAA-WNL<br>ngtdm-Contrast-Dose-MAA-WNL<br>glcm-MCC-Dose-MAA-Tumor<br>shape-MinorAxisLength-Dose-MAA-WNL<br>shape-LeastAxisLength-Dose-MAA-WNL<br>shape-VoxelVolume-Dose-MAA-WNL<br>ngtdm-Coarseness-Dose-MAA-WNL<br>shape-Maximum2DDiameterSlice-Dose-MAA-WNL
	Radiomics MAA-SPECT-CT-Clinical-MRMR	ngtdm-Strength-CT-MAA-Tumor<br>glcm-MaximumProbability-CT-MAA-WNL<br>shape-Maximum3DDiameter-CT-MAA-WNL<br>glszm-SizeZoneNonUniformityNormalized-CT-MAA-WNL<br>PVT<br>firstorder-10Percentile-SPECT-MAA-WNL<br>firstorder-Range-CT-MAA-NPL	shape_Maximum3DDiameter-CT-MAA-NPL<br>shape_MajorAxisLength-CT-MAA-WNL<br>Ascites<br>ngtdm_Coarseness-SPECT-MAA-WNL<br>gldm_DependenceNonUniformity-SPECT-MAA-Tumor<br>firstorder_RootMeanSquared-CT-MAA-NPL<br>shape_Maximum3DDiameter-SPECT-MAA-WNL	shape-Maximum2DDiameterColumn-CT-MAA-WNL<br>shape-Maximum3DDiameter-CT-MAA-WNL<br>ngtdm-Complexity-CT-MAA-WNL<br>shape-Maximum2DDiameterColumn-SPECT-MAA-WNL<br>glrlm-RunLengthNonUniformity-SPECT-MAA-Tumor<br>shape-Maximum2DDiameterRow-CT-MAA-Tumor<br>glszm-LargeAreaEmphasis-CT-MAA-NPL
^{90}Y	DVH-BED-ANOVA	Volume-WNL-ml<br>Y90-TNR-WNL<br>Y90-BED-HI-TL<br>Y90-BED-MAX-WNL-Gy<br>Y90-BED-Mean-TL-Gy	Volume-WNL-ml<br>Y90-BED-V30(ml)-NPL<br>Injected-Activity-Y90<br>Y90-BED-HI-TL<br>Y90-BED-MAX-TL-Gy	Volume-WNL-ml<br>Y90-BED-HI-TL<br>Y90-TNR-WNL<br>Y90-BED-V50(%) -WNL<br>Y90-BED-V205(%)-NPL
	DVH-BED-Dose-ANOVA	Volume-WNL-ml<br>Y90-TNR-WNL<br>Y90-BED-HI-TL<br>Y90-BED-MAX-WNL-Gy<br>Y90-TNR-NPL	Volume-WNL-ml<br>Volume-NPL-ml<br>Injected-Activity-Y90<br>Y90-Dose-Min-NPL-Gy<br>Y90-BED-HI-TL	Y90-Dose-HI-TL<br>Volume-WNL-ml<br>Y90-BED-HI-TL<br>Y90-TNR-WNL<br>Y90-Dose-Min-NPL-Gy
	DVH-BED-Dose-Relief	Volume-WNL-ml<br>Y90-TNR-NPL<br>Y90-BED-HI-TL<br>Y90-TNR-WNL<br>Y90-Dose-V400(%) -WNL	Volume-WNL-ml<br>Y90-BED-HI-TL<br>Y90-Dose-V400(%) -WNL<br>Volume-NPL-ml<br>Y90-BED-MAX-TL-Gy	Y90-Dose-HI-TL<br>Volume-WNL-ml<br>Y90-BED-HI-TL<br>Y90-BED-V120(%)-TL<br>Y90-Dose-Min-NPL-Gy
	Dosiomics BED-ANOVA	glcm-MCC-BED-Y90-Tumor<br>shape-MinorAxisLength-BED-Y90-WNL<br>glszm-GrayLevelNonUniformity-BED-Y90-WNL<br>shape-MajorAxisLength-BED-Y90-WNL<br>shape-Maximum2DDiameterColumn-BED-Y90-WNL	shape-MinorAxisLength-BED-Y90-WNL<br>shape-Maximum2DDiameterColumn-BED-Y90-WNL<br>shape-SurfaceArea-BED-Y90-WNL<br>shape-MinorAxisLength-BED-Y90-NPL<br>glrlm-RunLengthNonUniformity-BED-Y90-WNL	shape-MinorAxisLength-BED-Y90-WNL<br>shape-MeshVolume-BED-Y90-WNL<br>glrlm-RunLengthNonUniformity-BED-Y90-WNL<br>glcm-DifferenceVariance-BED-Y90-WNL<br>shape-Maximum2DDiameterSlice-BED-Y90-WNL
	Dosiomics BED-Kruskal	shape-MinorAxisLength-BED-Y90-WNL<br>glcm-MCC-BED-Y90-Tumor<br>shape-Maximum2DDiameterColumn-BED-Y90-WNL<br>shape-MajorAxisLength-BED-Y90-WNL<br>shape-SurfaceArea-BED-Y90-WNL	shape-MinorAxisLength-BED-Y90-WNL<br>shape-Maximum2DDiameterColumn-BED-Y90-WNL<br>shape-Maximum2DDiameterColumn-BED-Y90-NPL<br>glszm-GrayLevelNonUniformityNormalized-BED-Y90-Tumor<br>shape-Maximum3DDiameter-BED-Y90-WNL	shape-MinorAxisLength-BED-Y90-WNL<br>shape-MeshVolume-BED-Y90-WNL<br>glrlm-RunLengthNonUniformity-BED-Y90-WNL<br>firstorder-Maximum-BED-Y90-Tumor<br>glrlm-LowGrayLevelRunEmphasis-BED-Y90-Tumor
	Dosiomics BED-Clinical-ANOVA	Albumin<br>glcm-MCC-BED-Y90-Tumor<br>shape-MinorAxisLength-BED-Y90-WNL<br>glszm-GrayLevelNonUniformity-BED-Y90-WNL<br>shape-MajorAxisLength-BED-Y90-WNL	Ascites<br>shape-MinorAxisLength-BED-Y90-WNL<br>shape-Maximum2DDiameterColumn-BED-Y90-WNL<br>shape-SurfaceArea-BED-Y90-WNL<br>shape-MinorAxisLength-BED-Y90-NPL	shape-MinorAxisLength-BED-Y90-WNL<br>shape-MeshVolume-BED-Y90-WNL<br>glrlm-RunLengthNonUniformity-BED-Y90-WNL<br>glcm-DifferenceVariance-BED-Y90-WNL<br>shape-Maximum2DDiameterSlice-BED-Y90-WNL
	Dosiomics BED-Clinical-Kruskal	Albumin<br>shape-MinorAxisLength-BED-Y90-WNL<br>glcm-MCC-BED-Y90-Tumor<br>shape-Maximum2DDiameterColumn-BED-Y90-WNL<br>shape-MajorAxisLength-BED-Y90-WNL	shape-MinorAxisLength-BED-Y90-WNL<br>Ascites<br>shape-Maximum2DDiameterColumn-BED-Y90-WNL<br>shape-Maximum2DDiameterColumn-BED-Y90-NPL<br>glszm-GrayLevelNonUniformityNormalized-BED-Y90-Tumor	shape-MinorAxisLength-BED-Y90-WNL<br>shape-MeshVolume-BED-Y90-WNL<br>glrlm-RunLengthNonUniformity-BED-Y90-WNL<br>firstorder-Maximum-BED-Y90-Tumor<br>glrlm-LowGrayLevelRunEmphasis-BED-Y90-Tumor
	Dosiomics BED-Dose-ANOVA	glcm-MCC-BED-Y90-Tumor<br>shape-MinorAxisLength-Dose-Y90-WNL<br>glszm-GrayLevelNonUniformity-BED-Y90-WNL<br>shape-MajorAxisLength-Dose-Y90-WNL<br>shape-Maximum2DDiameterColumn-Dose-Y90-WNL	shape-MinorAxisLength-Dose-Y90-WNL<br>shape-Maximum2DDiameterColumn-Dose-Y90-WNL<br>shape-SurfaceArea-Dose-Y90-WNL<br>glrlm-RunLengthNonUniformity-Dose-Y90-WNL<br>glrlm-GrayLevelNonUniformity-Dose-Y90-NPL	shape-MinorAxisLength-Dose-Y90-WNL<br>shape-MeshVolume-Dose-Y90-WNL<br>glrlm-RunLengthNonUniformity-Dose-Y90-WNL<br>glcm-MCC-Dose-Y90-Tumor<br>glszm-SmallAreaEmphasis-Dose-Y90-WNL
	Dosiomics BED-Dose-Kruskal	shape-MinorAxisLength-Dose-Y90-WNL<br>glcm-MCC-BED-Y90-Tumor<br>shape-Maximum2DDiameterColumn-Dose-Y90-WNL<br>shape-MajorAxisLength-Dose-Y90-WNL<br>shape-SurfaceArea-Dose-Y90-WNL	shape-MinorAxisLength-Dose-Y90-WNL<br>shape-Maximum2DDiameterColumn-Dose-Y90-WNL<br>glrlm-GrayLevelNonUniformity-Dose-Y90-NPL<br>glszm-GrayLevelNonUniformityNormalized-BED-Y90-Tumor<br>glszm-GrayLevelNonUniformity-BED-Y90-WNL	shape-MinorAxisLength-Dose-Y90-WNL<br>glrlm-GrayLevelNonUniformity-Dose-Y90-WNL<br>shape-MeshVolume-Dose-Y90-WNL<br>glszm-GrayLevelVariance-Dose-Y90-Tumor<br>firstorder-Skewness-Dose-Y90-WNL
	Dosiomics BED-Dose-Clinical-ANOVA	Albumin<br>glcm-MCC-BED-Y90-Tumor<br>shape-MinorAxisLength-Dose-Y90-WNL<br>glszm-GrayLevelNonUniformity-BED-Y90-WNL<br>shape-MajorAxisLength-Dose-Y90-WNL	Ascites<br>shape-MinorAxisLength-Dose-Y90-WNL<br>shape-Maximum2DDiameterColumn-Dose-Y90-WNL<br>shape-SurfaceArea-Dose-Y90-WNL<br>glrlm-RunLengthNonUniformity-Dose-Y90-WNL	shape-MinorAxisLength-Dose-Y90-WNL<br>shape-MeshVolume-Dose-Y90-WNL<br>glrlm-RunLengthNonUniformity-Dose-Y90-WNL<br>glcm-MCC-Dose-Y90-Tumor<br>glszm-SmallAreaEmphasis-Dose-Y90-WNL
	Dosiomics BED-Dose-Clinical-Kruskal	Albumin<br>shape-MinorAxisLength-Dose-Y90-WNL<br>glcm-MCC-BED-Y90-Tumor<br>shape-Maximum2DDiameterColumn-Dose-Y90-WNL<br>shape-MajorAxisLength-Dose-Y90-WNL	shape-MinorAxisLength-Dose-Y90-WNL<br>Ascites<br>shape-Maximum2DDiameterColumn-Dose-Y90-WNL<br>glrlm-GrayLevelNonUniformity-Dose-Y90-NPL<br>glszm-GrayLevelNonUniformityNormalized-BED-Y90-Tumor	shape-MinorAxisLength-Dose-Y90-WNL<br>glrlm-GrayLevelNonUniformity-Dose-Y90-WNL<br>shape-MeshVolume-Dose-Y90-WNL<br>glszm-GrayLevelVariance-Dose-Y90-Tumor<br>firstorder-Skewness-Dose-Y90-WNL
	Dosiomics Dose-ANOVA	shape-MinorAxisLength-Dose-Y90-WNL<br>shape-MajorAxisLength-Dose-Y90-WNL<br>shape-Maximum2DDiameterColumn-Dose-Y90-WNL<br>shape-SurfaceArea-Dose-Y90-WNL<br>glcm-MCC-Dose-Y90-Tumor	shape-MinorAxisLength-Dose-Y90-WNL<br>shape-Maximum2DDiameterColumn-Dose-Y90-WNL<br>shape-SurfaceArea-Dose-Y90-WNL<br>glrlm-RunLengthNonUniformity-Dose-Y90-WNL<br>glrlm-GrayLevelNonUniformity-Dose-Y90-NPL	shape-MinorAxisLength-Dose-Y90-WNL<br>shape-MeshVolume-Dose-Y90-WNL<br>glrlm-RunLengthNonUniformity-Dose-Y90-WNL<br>glcm-MCC-Dose-Y90-Tumor<br>glszm-SmallAreaEmphasis-Dose-Y90-WNL

Dosiomics Dose-Kruskal	shape-MinorAxisLength-Dose-Y90-WNL<br>shape-Maximum2DDiameterColumn-Dose-Y90-WNL<br>shape-MajorAxisLength-Dose-Y90-WNL<br>shape-SurfaceArea-Dose-Y90-WNL<br>glcm-MCC-Dose-Y90-Tumor	shape-MinorAxisLength-Dose-Y90-WNL<br>shape-Maximum2DDiameterColumn-Dose-Y90-WNL<br>glrlm-GrayLevelNonUniformity-Dose-Y90-NPL<br>shape-Maximum3DDiameter-Dose-Y90-WNL<br>shape-SurfaceArea-Dose-Y90-WNL	shape-MinorAxisLength-Dose-Y90-WNL<br>glrlm-GrayLevelNonUniformity-Dose-Y90-WNL<br>shape-MeshVolume-Dose-Y90-WNL<br>glszm-GrayLevelVariance-Dose-Y90-Tumor<br>firstorder-Skewness-Dose-Y90-WNL
Dosiomics Dose-Relief	shape-MinorAxisLength-Dose-Y90-WNL<br>glcm-MCC-Dose-Y90-Tumor<br>shape-SurfaceArea-Dose-Y90-WNL<br>ngtdm-Busyness-Dose-Y90-WNL<br>glrlm-GrayLevelNonUniformityNormalized-Dose-Y90-NPL	glszm-ZoneEntropy-Dose-Y90-WNL<br>shape-Maximum2DDiameterColumn-Dose-Y90-WNL<br>shape-MinorAxisLength-Dose-Y90-WNL<br>glcm-SumEntropy-Dose-Y90-NPL<br>firstorder-Range-Dose-Y90-NPL	firstorder-Range-Dose-Y90-Tumor<br>firstorder-Maximum-Dose-Y90-Tumor<br>glcm-MCC-Dose-Y90-Tumor<br>shape-MinorAxisLength-Dose-Y90-WNL<br>firstorder-90Percentile-Dose-Y90-Tumor
Dosiomics Dose-Clinical-ANOVA	Albumin<br>shape-MinorAxisLength-Dose-Y90-WNL<br>shape-MajorAxisLength-Dose-Y90-WNL<br>shape-Maximum2DDiameterColumn-Dose-Y90-WNL<br>shape-SurfaceArea-Dose-Y90-WNL	Ascites<br>shape-MinorAxisLength-Dose-Y90-WNL<br>shape-Maximum2DDiameterColumn-Dose-Y90-WNL<br>shape-SurfaceArea-Dose-Y90-WNL<br>glrlm-RunLengthNonUniformity-Dose-Y90-WNL	shape-MinorAxisLength-Dose-Y90-WNL<br>shape-MeshVolume-Dose-Y90-WNL<br>glrlm-RunLengthNonUniformity-Dose-Y90-WNL<br>glcm-MCC-Dose-Y90-Tumor<br>glszm-SmallAreaEmphasis-Dose-Y90-WNL
Dosiomics Dose-Clinical-Kruskal	Albumin<br>shape-MinorAxisLength-Dose-Y90-WNL<br>shape-Maximum2DDiameterColumn-Dose-Y90-WNL<br>shape-MajorAxisLength-Dose-Y90-WNL<br>shape-SurfaceArea-Dose-Y90-WNL	shape-MinorAxisLength-Dose-Y90-WNL<br>Ascites<br>shape-Maximum2DDiameterColumn-Dose-Y90-WNL<br>glrlm-GrayLevelNonUniformity-Dose-Y90-NPL<br>shape-Maximum3DDiameter-Dose-Y90-WNL	shape-MinorAxisLength-Dose-Y90-WNL<br>glrlm-GrayLevelNonUniformity-Dose-Y90-WNL<br>shape-MeshVolume-Dose-Y90-WNL<br>glszm-GrayLevelVariance-Dose-Y90-Tumor<br>firstorder-Skewness-Dose-Y90-WNL
Radiomics-SPECT-ANOVA	shape-MinorAxisLength-SPECT-Y90-WNL<br>ngtdm-Coarseness-SPECT-Y90-WNL<br>shape-MajorAxisLength-SPECT-Y90-WNL<br>shape-Maximum2DDiameterColumn-SPECT-Y90-WNL<br>shape-SurfaceArea-SPECT-Y90-WNL	shape-MinorAxisLength-SPECT-Y90-WNL<br>shape-Maximum2DDiameterColumn-SPECT-Y90-WNL<br>shape-SurfaceArea-SPECT-Y90-WNL<br>shape-Maximum3DDiameter-SPECT-Y90-NPL<br>shape-MinorAxisLength-SPECT-Y90-NPL	glcm-InverseVariance-SPECT-Y90-WNL<br>glcm-Idmn-SPECT-Y90-WNL<br>glcm-Idmn-SPECT-Y90-NPL<br>shape-MinorAxisLength-SPECT-Y90-WNL<br>Shape-VoxelVolume-SPECT-Y90-WNL
Radiomics-SPECT-Kruskal	shape-MinorAxisLength-SPECT-Y90-WNL<br>shape-Maximum2DDiameterColumn-SPECT-Y90-WNL<br>shape-MajorAxisLength-SPECT-Y90-WNL<br>shape-SurfaceArea-SPECT-Y90-WNL<br>ngtdm-Coarseness-SPECT-Y90-WNL	shape-MinorAxisLength-SPECT-Y90-WNL<br>shape-Maximum2DDiameterColumn-SPECT-Y90-WNL<br>shape-Maximum3DDiameter-SPECT-Y90-WNL<br>shape-SurfaceArea-SPECT-Y90-WNL<br>shape-Maximum3DDiameter-SPECT-Y90-NPL	shape-MinorAxisLength-SPECT-Y90-WNL<br>glcm-InverseVariance-SPECT-Y90-WNL<br>gldm-DependenceVariance-SPECT-Y90-WNL<br>shape-SurfaceArea-SPECT-Y90-WNL<br>shape-VoxelVolume-SPECT-Y90-WNL
Radiomics-SPECT-Clinical-ANOVA	Albumin<br>shape-MinorAxisLength-SPECT-Y90-WNL<br>ngtdm-Coarseness-SPECT-Y90-WNL<br>shape-MajorAxisLength-SPECT-Y90-WNL<br>shape-Maximum2DDiameterColumn-SPECT-Y90-WNL	Ascites<br>shape-MinorAxisLength-SPECT-Y90-WNL<br>shape-Maximum2DDiameterColumn-SPECT-Y90-WNL<br>shape-SurfaceArea-SPECT-Y90-WNL<br>shape-Maximum3DDiameter-SPECT-Y90-NPL	glcm-InverseVariance-SPECT-Y90-WNL<br>glcm-Idmn-SPECT-Y90-WNL<br>glcm-Idmn-SPECT-Y90-normal-lobe<br>shape-MinorAxisLength-SPECT-Y90-WNL<br>shape-MeshVolume-SPECT-Y90-WNL
Radiomics-SPECT-Clinical-Kruskal	Albumin<br>shape-MinorAxisLength-SPECT-Y90-WNL<br>shape-Maximum2DDiameterColumn-SPECT-Y90-WNL<br>shape-MajorAxisLength-SPECT-Y90-WNL<br>shape-SurfaceArea-SPECT-Y90-WNL	shape-MinorAxisLength-SPECT-Y90-WNL<br>Ascites<br>shape-Maximum2DDiameterColumn-SPECT-Y90-WNL<br>shape-Maximum3DDiameter-SPECT-Y90-WNL<br>shape-SurfaceArea-SPECT-Y90-WNL	shape-MinorAxisLength-SPECT-Y90-WNL<br>glcm-InverseVariance-SPECT-Y90-WNL<br>gldm-DependenceVariance-SPECT-Y90-WNL<br>shape-MeshVolume-SPECT-Y90-WNL<br>shape-SurfaceArea-SPECT-Y90-WNL

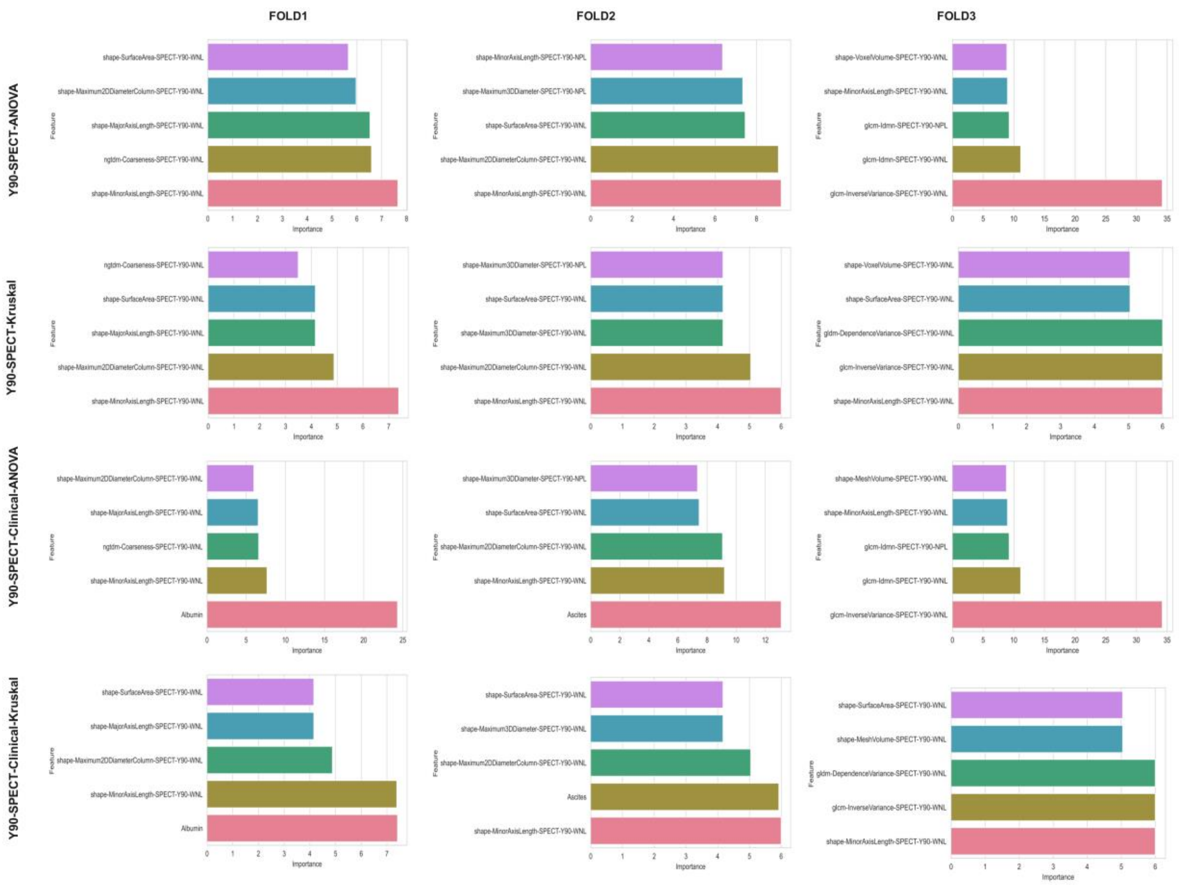

Figure 7. Feature importances of selected features from well-performing ^{90}Y-Radiomics models. The feature importances are displayed for each fold.

Figure 8. Feature importances of selected features from well-performing MAA-DVH, MAA-Radiomics and ^{90}Y-DVH and ^{90}Y-Dosiomics-bsed models. The selected features are displayed for each fold.

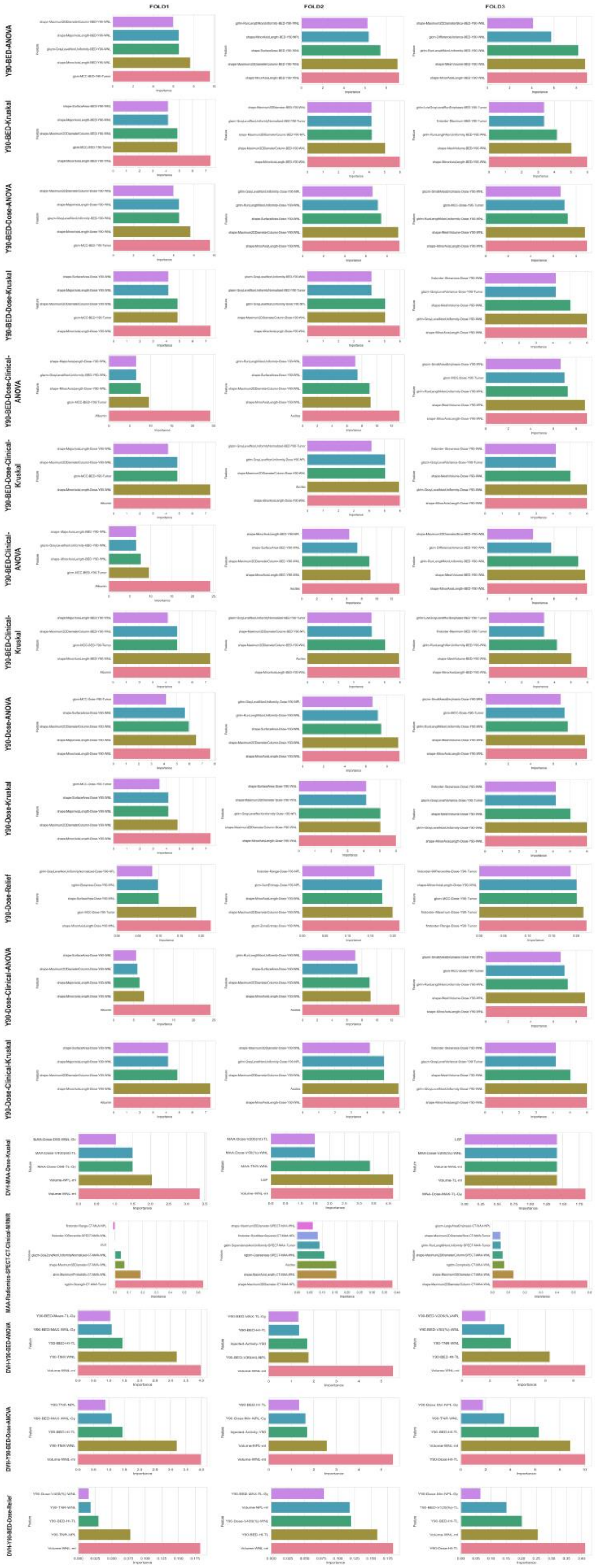

Supplement: Supplementary file 1 — Supplementary file1 (PDF 2191 KB) [file 11307_2025_1992_MOESM1_ESM.pdf]
